# Comparative transcriptomics and metabolomics in a rhesus macaque drug administration study

**DOI:** 10.3389/fcell.2014.00054

**Published:** 2014-10-08

**Authors:** Kevin J. Lee, Weiwei Yin, Dalia Arafat, Yan Tang, Karan Uppal, ViLinh Tran, Monica Cabrera-Mora, Stacey Lapp, Alberto Moreno, Esmeralda Meyer, Jeremy D. DeBarry, Suman Pakala, Vishal Nayak, Jessica C. Kissinger, Dean P. Jones, Mary Galinski, Mark P. Styczynski, Greg Gibson

**Affiliations:** ^1^Center for Integrative Genomics, School of Biology, Georgia Institute of TechnologyAtlanta, GA, USA; ^2^School of Chemical and Biomolecular Engineering, Georgia Institute of TechnologyAtlanta, GA, USA; ^3^Division of Pulmonary, Allergy and Critical Care Medicine, Department of Medicine, School of Medicine, Emory UniversityAtlanta, GA, USA; ^4^Emory Vaccine Center and Yerkes National Primate Research Center, Emory UniversityAtlanta, GA, USA; ^5^Division of Infectious Diseases, Department of Medicine, Emory UniversityAtlanta, GA, USA; ^6^Center for Topical and Emerging Global Diseases, University of GeorgiaAthens, GA, USA; ^7^Institute of Bioinformatics, University of GeorgiaAthens, GA, USA

**Keywords:** pyrimethamine, bone marrow, peripheral blood, axes of variation, bayesian network inference, principal component analysis (PCA)

## Abstract

We describe a multi-omic approach to understanding the effects that the anti-malarial drug pyrimethamine has on immune physiology in rhesus macaques (*Macaca mulatta*). Whole blood and bone marrow (BM) RNA-Seq and plasma metabolome profiles (each with over 15,000 features) have been generated for five naïve individuals at up to seven timepoints before, during and after three rounds of drug administration. Linear modeling and Bayesian network analyses are both considered, alongside investigations of the impact of statistical modeling strategies on biological inference. Individual macaques were found to be a major source of variance for both omic data types, and factoring individuals into subsequent modeling increases power to detect temporal effects. A major component of the whole blood transcriptome follows the BM with a time-delay, while other components of variation are unique to each compartment. We demonstrate that pyrimethamine administration does impact both compartments throughout the experiment, but very limited perturbation of transcript or metabolite abundance was observed following each round of drug exposure. New insights into the mode of action of the drug are presented in the context of pyrimethamine's predicted effect on suppression of cell division and metabolism in the immune system.

## Introduction

The Malaria Host-Pathogen Interaction Center (MaHPIC) has initiated a systems biology program to understand the course of events and mechanistic processes that occur in the biology of infected non-human primates (NHPs) and *Plasmodium* parasites over the course of malaria episodes. The long-term goal is to advance the development of interventions for this major global parasitic disease (WHO World Malaria Report, 2013). This research program investigates how NHP-infective species of *Plasmodium* that model human malaria caused by *P. falciparum* and *P. vivax* elicit various host responses, develop immunity, adopt immune-avoidance strategies, and cope with anti-malarial drugs (Galinski et al., 2013; Wright and Rayner, 2014). We are integrating diverse data types, including transcriptomics, metabolomics, lipidomics, proteomics, and innate and adaptive immune profiles and performing cross-species comparisons with multiple different host-parasite infection model combinations. There are many gaps in knowledge relating to *Plasmodium* infections including the immune response, the mechanisms of malaria pathogenesis and multiorgan dysfunction, the adverse impact on the bone marrow (BM) progenitors and the dynamics of co-infections (Hafalla et al., 2011; Schwenk and Richie, 2011; Frevert and Nacer, 2013; Stanisic et al., 2013). The NHP models being studied enable more rigorous experimentation and in-depth analyses than are possible from direct investigations in humans (Deye et al., 2012; Tachibana et al., 2012; Moreno et al., 2013) and they are well-suited for systems biology approaches.

In this study, we establish logistics and procedures that lay the foundation for studies of rhesus macaques (*Macaca mulatta*) inoculated with infectious *Plasmodium* parasites, following which intermittent antimalarial drug intervention may be required. The data presented here serve as pilot data, with inoculations consisting of Anopheline mosquito salivary gland preparations lacking sporozoites, and the analyses begin to show how multiple diverse datasets can be integrated. We present multi-omic data analyses using top-down approaches to the integration of RNA-Seq derived transcriptome data from the BM and peripheral blood (PB), as well as plasma metabolite data, and complete blood cell count (CBC) parameters. These data types were obtained during a 100-day period, at specific timepoints before and after pyrimethamine administration to five rhesus macaques.

**Figure 1:**
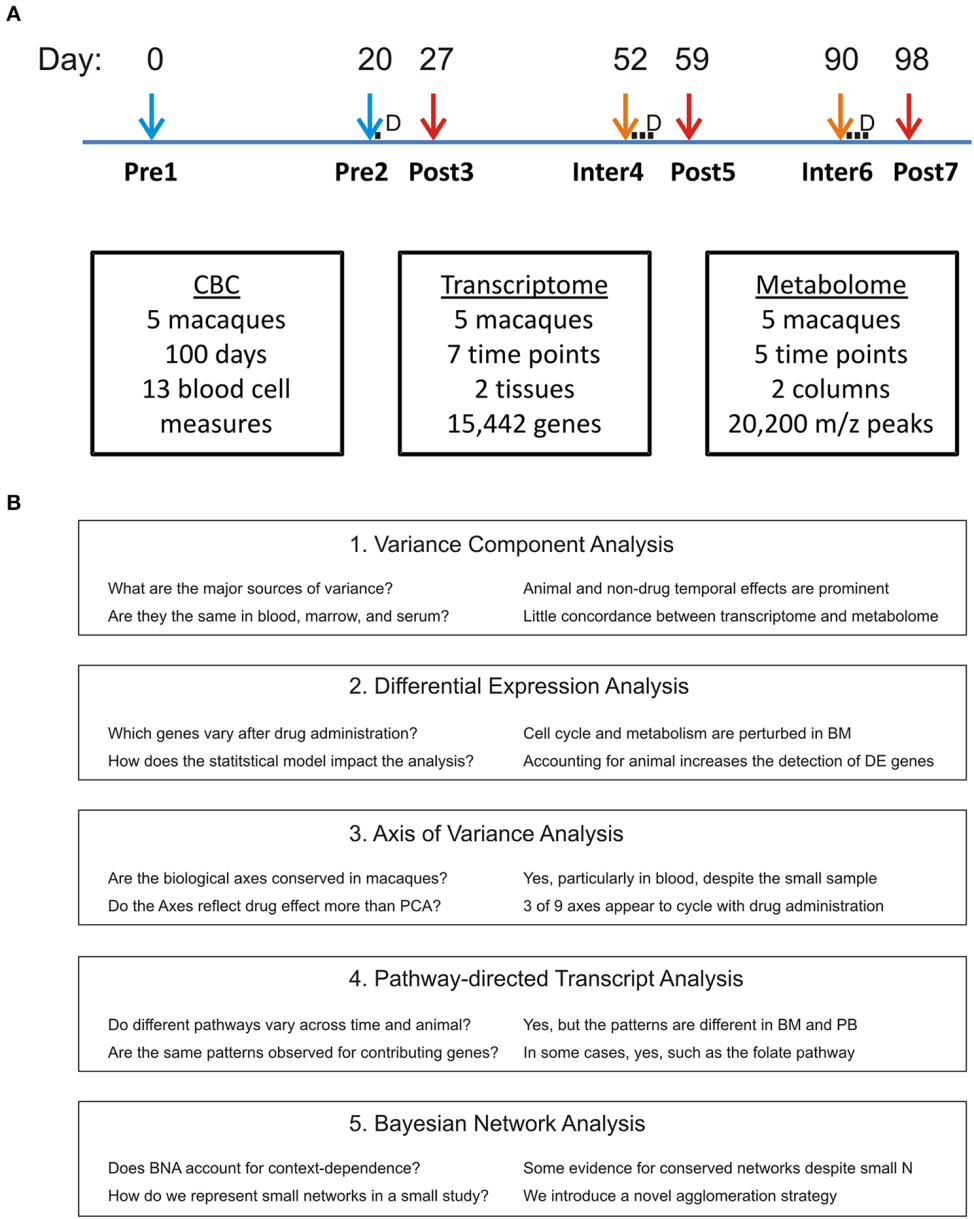
**Experimental Design. (A)** Five macaques were each delivered a sub-curative dose of pyrimethamine at Day 21, and 3-day curative doses commencing at Days 52 and 90, in each case immediately following peripheral blood sampling. This results in two pre-drug, three post-drug, and two inter-drug treatments as indicated. Metabolome data was not generated for the first two timepoints. **(B)** Flow of analytical approaches including major questions asked and inferences drawn.

By top-down integration, we mean statistical and machine-learning strategies that are naïve to the known biochemical annotation of the transcript and metabolite features (Bang et al., 2008; Giuliani et al., 2014). Our approach is to use principal components analysis (PCA) to describe the major sources of variance (among individual animals or temporal) in each data type, and then to seek correlations between the major components across data types (Boedigheimer et al., 2008). We perform standard differential gene expression analysis, also asking how the statistical modeling strategy and data reduction influence identification of drug-responsive genes, and employ gene set enrichment analysis to identify pathways of interest. In an attempt to overcome the limitations of orthogonal PCA, particularly in the context of a relatively small experiment, we ask whether biologically derived axes of variation that are known to consistently capture PB variation in humans, are conserved in macaques and covary with drug treatment. A bottom-up strategy, starting with known cellular and biochemical pathways from the Kyoto Encyclopedia of Genes and Genomes (KEGG: Kanehisa and Goto, 2000; Kanehisa et al., 2014), is contrasted and used to help draw more inferences about the physiological impact of pyrimethamine particularly on the BM. Finally, Bayesian network analysis (Bumgarner and Yeung, 2009; Pei and Shin, 2012) is also applied as an orthogonal approach with promise for overcoming the conditional dependence of the transcriptome and metabolome in a dataset with high dimensionality and a small number of samples. The work flow is shown in Figure 1 including the questions posed by each mode of analysis and the major conclusions.

The null hypotheses are first that neither host nor drug administration impact gene expression, and second that the major variance components of the BM and PB transcriptomes and plasma metabolome are uncorrelated. Transcriptome data was collected by RNA-Seq (Wilhelm and Landry, 2009) with a mean of 40 million paired-end reads for each of 35 BM and 35 PB samples studied (five macaques each with seven collection timepoints), focusing on transcript abundance for ~15,000 genes. Metabolome data was collected by Orbitrap mass spectrometry following liquid chromatography (Jones et al., 2012; Soltow et al., 2013) on two different columns (AE and C18) with ~6000 and ~14,500 m/z features, respectively. Our expectation was that drug administration would have global effects on each of the four omic measures (BM and PB transcriptomes, and AE and C18 generated metabolomes), and that among-individual differences would be relatively minor. However, we had no pre-conception of the fraction of genes that would be differentially expressed, or of the degree of correspondence we would find between the transcriptomes and metabolome. Since the PB consists of cells generated in the BM, we expected a temporal delay between these two compartments with considerable overlap in variance components, which would also reflect differences in the counts of major blood cell types obtained by standard CBC analysis. Herein we quantify departures from each of these expectations as well as a general failure to reject the null hypothesis that the blood transcriptomes and metabolomes are uncorrelated, and discuss the implications for the mode of action of pyrimethamine.

## Materials and methods

### Experimental design

The experimental design of this experiment involving rhesus macaques (*Macaca mulatta*) was approved by the Emory University Institutional Animal Care and Use Committee (IACUC) and is as follows. Five males (*RCs13, RWr13, RUn13, RZe13*, and *RTi13*) approximately 2 years of age were injected intravenously with a preparation of *Anopheles dirus* salivary gland material (prepared similarly to how infectious *Plasmodium* sporozoites would be purified; Kennedy et al., 2012) and then profiled for clinical and omic measurements over the course of a 100-day experiment. The animals were moved into experimental pair housing (RCs13/RWr13 and RUn13/RZe13) 10 days prior to the baseline sampling point at Day 0, namely timepoint 1 (TP1). The fifth macaque (RTi13) was housed alone. Capillary blood samples collected daily from ear pricks into EDTA-tubes were used to obtain complete blood cell counts (CBCs), with the exception of days 51 to 53 when an equipment failure occurred. On days 21, 27, 52, 59, 90, and 98, PB and BM samples were collected comprising TPs 2-7. These collections, and that of TP1, were taken under chemical restraint with ketamine delivered intramuscularly at 10 mg/kg. This dissociative anesthetic has a short elimination half-life (20–40 min) and, to our knowledge, has no known drug interaction with pyrimethamine. The experimental design does not, however, allow for distinguishing the effects of the drug or anesthetic. BM aspirates were obtained from the right or left iliac crests in an alternating manner for consecutive timepoints and performed using 18G needles. Immediately after collection BM samples were transferred into Vacutainer EDTA tubes. PB samples were collected from the femoral artery into Vacutainer EDTA tubes. The transcriptomes and metabolomes were interrogated at seven (TP1-7) and five (TP3-7) timepoints, respectively, as shown in Figure 1. Pyrimethamine (Sigma-P7771) was delivered (1 mg/kg) intramuscularly once on day 20, and for 3 successive days starting at days 52 and 90 (TP2, 4, and 6), corresponding to predicted periods for sub-curative and curative experimental treatment regimens for malaria infection of macaques.

### Library preparation for RNA-Seq

BM (1 ml) was collected into 1.5 ml tubes with EDTA, and the mononuclear cells were purified by density gradient centrifugation on Lymphoprep (Stem Cell Technologies) solution and preserved in RLT buffer (Qiagen) to stabilize mRNA. Whole blood (3 ml) was collected in Tempus tubes (Applied Biosystems) which preserve mRNA; these samples include erythrocytes, platelets and granulocytes, and mononuclear lymphocytes. RNA was extracted from the BM samples using Qiagen RNEasy Mini-Plus kits following the manufacturer-recommended procedures, and from PB samples using Tempus-Spin RNA isolation kits (Life Technologies). The quality of all RNA samples was confirmed using a Bioanalyzer, with an RNA Integrity Number (RIN; Schroeder et al., 2006) greater than 8 recorded for all samples.

Approximately 1 μg of total RNA per sample was converted to double-stranded cDNA using poly-A beads to enrich for mRNA, and Illumina TruSeq Stranded mRNA Sample Prep kits to generate strand-specific libraries. As a quality control, 96 spike-in RNAs of known concentration and GC proportions (ERCC Spike-In Control, Life Technologies; Devonshire et al., 2010) were added to constitute approximately 1% of the total RNA for each library. Adapters were ligated to facilitate 3-plex sequencing on an Illumina HiSeq2000 at the Yerkes National Primate Center Genomics Core, aiming for 80 million paired-end 100 base pair (bp) reads per library. Average insert sizes were in the range of 300–400 bp.

### Short read mapping and gene expression quantification

To quantify gene expression, the RNA-Seq reads were mapped to an early version of a new assembly of the rhesus macaque (MacaM assembly, Version 4.0, GenBank accession number PRJNA214746 ID: 214746, created by Aleksey Zimin at the University of Maryland, Rob Norgren at the University of Nebraska Medical Center, and their colleagues) using Tophat2 (Trapnell et al., 2012; Kim et al., 2013). Default options were used with the exception that the command—library-type fr-secondstrand was invoked since the reads were generated using a stranded library preparation method. This allowed us to differentiate between sense and antisense transcripts. Rob Norgren and his colleagues also provided a GTF file (version 4.12) of the annotated MacaM assembly indicating the exon boundaries of rhesus genes that was used in our transcriptome analyses to improve the mapping accuracy across splice junctions. Only reads that map to a single location in the genome were included, to ensure high-confidence mapping. All downstream analyses were performed at the level of annotated gene: this study does not consider exon-specific or transcript isoform relative abundance. Transcription was detected for 15,442 genes. The dataset has been deposited to the Gene Expression Omnibus archive (GEO) under accession number GSE58340.

Several quality control steps were used to verify the reliability of the data: linear correlation of estimated abundance of ERCC spike-in controls with known concentration; confirmation of 99.9% strand-specificity of the controls; less than 0.1% control fusion transcripts; and absence of 3′ bias in the controls was confirmed with RSeqC v2.3.8 software (http://rseqc.sourceforge.net; Wang et al., 2012). Transcript abundance levels were inferred using HTSeq v0.5.4p5 (http://www-huber.embl.de/users/anders/HTSeq/doc; Anders et al., 2014). HTSeq takes the short-read mapping.bam file from tophat2 and the gene annotation file which contains the locations of all annotated genes. Since some libraries were sequenced more deeply than others, the libraries were normalized before determining differential gene expression using the gene level expression files with the default parameters of DESeq version 1.10.1 (http://www.bioconductor.org/packages/release/bioc/html/DESeq.html; Anders and Huber, 2010).

### Metabolomic feature quantification

High resolution metabolomics (m/z range 85–2000) was performed using a liquid chromatography/mass spectrometry (LC/MS) approach on a Thermo Orbitrap-Velos Mass Spectrometer (Thermo Fisher, San Diego, CA) via positive-ion electrospray ionization (ESI). Two different columns were used for the LC separation stage: C18 and anion exchange (AE). Each distinct biological sample was run in triplicate in order to ensure high reliability of the data, with randomization within batches (Soltow et al., 2013). MS peaks were called using xMSanalyzer v1.3.2 (Uppal et al., 2013) with apLCMS v5.9.4 (Yu et al., 2009). Standard quality control measures were performed, such that features with greater than 30% missing values were removed from the analysis. Since the frequency distributions of all samples were comparable, no additional normalization was performed, but an abundance cutoff of 256 peak area units was adopted and all features below this were excluded. All downstream analyses utilized the median values of three technical replicate samples, namely a single measure per biological sample. The AE and C18 columns generated 5861 and 14,339 m/z and retention time features respectively, the majority of which are either not yet annotated or have ambiguous annotation to multiple possible organic compounds. The m/z features are thought to include the majority of known components of central metabolism, as well as xenobiotics.

### Statistical analysis

After data normalization, the transcriptome and metabolome levels were log-2-transformed and imported into JMP Genomics (version 6.0, SAS Institute, Cary, NC). The log-2 transformation was performed both to ensure that the data is more normally distributed and to facilitate simple comparison of the magnitude of differential expression in a symmetrical manner with respect to up- and down-regulation, as is standard in microarray analysis: plus or minus 1 unit corresponds to a 2-fold change for each of the datasets. To determine how much of the variance in each of our datasets is explained by our two measured factors (animal and timepoint), we performed a principal components (PC)-variance component analysis using JMP v6.0 (SAS) for the transcriptomes, metabolomes, and the CBC data (Boedigheimer et al., 2008). This consists of generation of all PC explaining up to 90% of the total variance (12–15 for the transcriptomes and ~30 for the metabolomes), regressing each PC on “animal” or “timepoint,” and generating a weighted average of the squared correlation coefficient (percent variance explained) across all of the PC scores. Since the low abundance features for metabolomics and transcriptomics both have high coefficients of variation, we set thresholds of 5 log2 units for transcripts and 17 log2 units for metabolites based simply on visual inspection of plots of the coefficient of variance against average abundance. After estimating the effect of lower-abundance features on the variance components, they were removed for all downstream analyses. No attempt was made to optimize the threshold or systematically evaluate its impact, but the major conclusions of the study are unlikely to be affected.

To assess whether the major PC capture similar aspects of the data, the first 10 PC were calculated for the four omics datasets using JMP. All 780 pairwise correlations of these PC values were determined, and a Bonferroni multiple comparison adjustment was used to assess the significance of each pair of PC. Exploratory partial least square regression analyses were also performed with MixOmics (González et al., 2012; http://cran.r-project.org/web/packages/mixOmics/index.html) in an attempt to select variables that co-vary, but did not reveal significant associations.

### Blood informative transcript (BIT) axes

In addition to PCA, we employed a second method, blood informative transcript (BIT) axes analysis (Preininger et al., 2013). Briefly, 10 highly co-regulated transcripts in blood (the BIT) capture each of 9 common axes of variation that are observed in all human PB gene expression datasets. PC1 for each of these 9 sets of 10 transcripts provide Axis scores for each individual sample, and were generated independently for both the PB and BM samples, using the normalized expression data. We then examined the dynamics of the axes scores (or their residuals after fitting “Animal”) over time and used ANOVA to evaluate differences among the timepoints or animals.

### Differential gene expression

The next step in our analysis was the identification of genes that are differentially expressed across the experimental conditions (Soneson and Delorenzi, 2013). For between-TP differences, an ANOVA was performed on each transcript separately using “animal” as a random effect with five levels and “timepoint” with seven levels, or “drug” with three levels as the fixed effect. For the drug exposure factor, we define our three experimental conditions as before drug exposure (pre-drug; TP1 and TP2), 7 days after the most recent dose (post-drug; TP3, TP5, and TP7), and 30 days after most recent dose and immediately before the next dose (inter-drug; TP4 and TP6), as shown in Figure 1. A Benjamini-Hochberg false discovery rate cutoff of 5% was used to define differentially expressed genes. These were examined using hierarchical clustering of the standardized least squares means, and volcano plots of significance against fold difference between specific conditions (Wolfinger et al., 2001). The significantly differentially expressed genes are reported as a Supplementary flat file that consists of a list of gene names with their corresponding F-statistics at http://www.cig.gatech.edu/supplementary-data.

Gene set enrichment analyses were performed using pre-existing human gene set annotations from the Broad Institute (Subramanian et al., 2005), considering that the majority of known genes in the macaque genome have very closely related syntenic human orthologs (Zhang et al., 2014). We used the ranked gene list method of GSEA v2.0.14 (http://www.broadinstitute.org/gsea/index.jsp) to perform the contrast of interest (pre-vs.-[post plus inter] drug treatment), testing for enrichment of *t*-statistics in KEGG pathways and/or GO terms. Gene sets with a nominal *p* < 0.001 and an FDR *q* < 5% were considered as significant per the recommendations of the GSEA software manual. Default parameters were used, excluding gene sets with more than 500 or fewer than 20 genes.

### Bayesian network analysis

For the Bayesian network analyses, only the 1000 most differentially expressed genes (largest F-ratios for the Drug effect) or 500 metabolites were used, so as to ensure computational tractability of the clustering software while incorporating biologically relevant genes. The transcript abundance measures were the residuals after fitting “animal” in the ANOVA to remove this large overall source of variance, while raw median metabolite abundance measures were used based on the relatively small contribution of animal and timepoint to the variance. Custom scripts were written in MATLAB and R to perform quality threshold clustering (Heyer et al., 1999; De Smet et al., 2002) on the mean-centered expression values, namely the residuals after fitting “animal” to each gene. A *d* = 0.3 cluster similarity threshold was employed as suggested by Heyer et al. (1999). Since the data are normalized and since that similarity threshold is based on a specific type of correlation metric, it is reasonable to expect that such a value may be an excellent starting point across transcriptomic studies. We also performed small perturbations of the cluster similarity threshold and found that the main differences were in the merging portions of some smaller clusters into some bigger clusters. The number and membership of the larger clusters (used for analysis here) remained similar (data not shown). Only clusters with at least 10 transcripts (or metabolites) were retained for further analysis. Significance and robustness of these clusters were assessed via permutation tests.

Since robust and accurate Bayesian network inference is typically very difficult with only 35 observations (five animals and seven timepoints), we treated genes within clusters as separate observations of those clusters. We ranked each gene relative to its correlation with the centroid of the cluster across all 35 samples (25 for metabolites since TP1 and TP2 were missing) and then concatenated the top 10 genes into a list of 350 (250) observations for each of 26 transcript and four metabolite clusters that satisfied the clustering criteria. We chose to use just the top 10 genes so as to ensure that each node in the network has the same number of observations (no missing data) and because Bayesian network inference benchmarking literature has shown that having a few hundred observations can provide reasonably robust inference. The selection of 10 genes thus balances robustness with the number of different clusters that can be analyzed, as increasing that threshold necessarily eliminates more clusters. Next, the data was discretized using a mutual information content-preserving algorithm (Hartemink, 2001), as the Bayesian network analysis is expected to be more robust for discrete data. Briefly, for each variable, observations are stepwise coalesced into discretized bins such that the loss in mutual information content between that variable and all other variables is minimized. The estimated elbow-point in the remaining mutual information as a function of the number of discretization levels was then selected as the desired number of levels (seven for transcriptional and five for metabolite data). Networks were subsequently generated using the Sparse Candidate Algorithm (Friedman et al., 1999) of Causal Explorer in MATLAB (http://www.dsl-lab.org/causal_explorer; Aliferis et al., 2003). The most robust connections between clusters were identified using subsampling and permutation tests. We used three shuffled datasets, within which the order of the 10 genes in each cluster was permuted independently, to minimize the possibility of over-fitting the available data: if the 10 genes are good representatives of the cluster, then there should not be much mutual information based solely on a given gene in one cluster being compared to a specific gene in another cluster, and so the most robust edges (and least likely to be due to over-fitting) are the ones that occur in multiple shuffled datasets. For each of these shuffled datasets we assessed the sensitivity of the inference method to perturbations in the amount of available data by performing network inference with 90% subsampling of 1000 replicates of the dataset.

To assess whether the BM clusters are valid in the PB, the PB data for each of the clusters was used to determine their centroids. The distribution of Pearson correlation coefficients for each member of the cluster to the centroid was calculated. These distributions were compared to analogous calculations for random samples of the same number of genes for each cluster, using a one-tailed Kolmogorov–Smirnov test.

To assess whether the BM clusters form a network in the PB, we used the same 26 clusters from the BM data, identified the 10 genes closest to the centroid using the PB data for each cluster, and used concatenated gene data for each cluster to generate new Bayesian networks. To evaluate whether there are interactions between transcriptomic and metabolic networks, we repeated the Bayesian network inference using the 26 BM transcript clusters and four plasma metabolite clusters from the C18 column MS data analyzed with essentially the same pipeline. The same methods as described above were used to form this integrated network, except that for the transcriptional data only the five timepoints corresponding to the metabolomics timepoints were kept, the data discretization was performed jointly on the combined datasets, and metabolomics data was unit normalized.

## Results

### Variance components of omic data

The experimental design consisted of a 100 day mock-infection cycle of *M. mulatta* that follows a similar time course as will be used for a series of *Plasmodium* infections in later MaHPIC studies (Figure 1). Each of five monkeys was transferred, in two pairs and a single, to indoor cages at the Yerkes National Primate Research Center in Atlanta, Georgia, 10 days prior to the baseline (TP1) PB and BM draws. The second timepoint (TP2) samples were taken 20 days later, immediately prior to administration of the anti-malarial drug pyrimethamine for the first time and 7 days before sampling of the first post-Drug timepoint (TP3). After a further 3 weeks, a second round of drug treatment immediately followed the TP4 sampling. Consequently, TP1 and TP2 represent pre-drug samples that nevertheless differ in their gene expression and metabolic profiles, we suspect due to an acclimation period of each animal in the new experimental environment. TP3, TP5, and TP7 represent post-drug samples, and TP4 and TP6 represent inter-drug samples. Plasma metabolites were only profiled following the drug administration period (TP3-TP7), but CBCs were generated for all timepoints except TP4.

**Figure 2:**
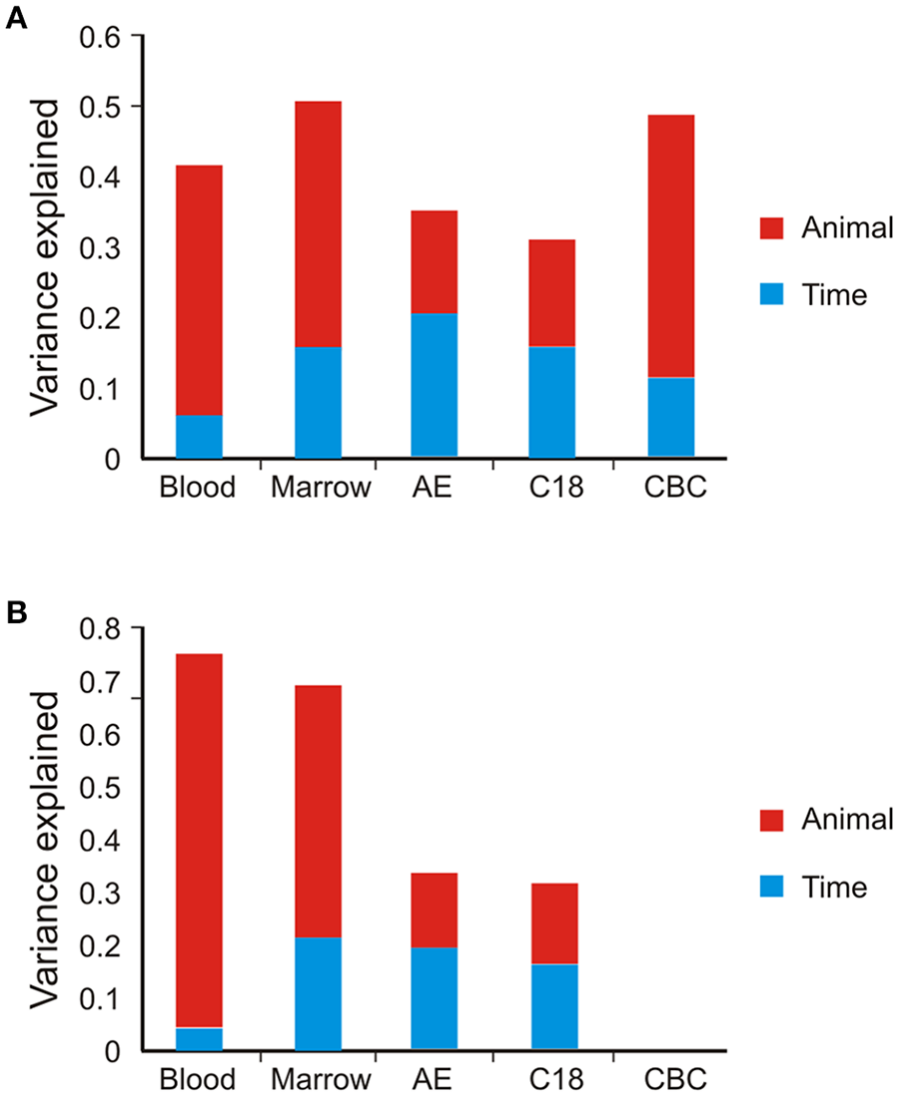
**Principal component variance component analyses**. Bar graphs show the weighted average contribution of among animal (red) and among timepoint (blue) variance to gene expression, metabolite, and complete blood counts. **(A)** Full data set. **(B)** Reduced dataset after removal of low abundance transcripts or metabolites.

Our first objective was to define the variance components of gene expression and metabolite abundance, namely the contributions of among animal and among timepoint differences to the overall variance. This was accomplished by generating the PC that collectively account for 90% of the variance of each omic data type and computing the weighted average of the variance of each PC explained by animal or timepoint. The variance explained by each PC is shown in Table 1 and typically ranges from 25% for PC1 to less than 3% for PC5 (subsequent PC contribute too little to the overall variance to significantly impact the evaluation of contributions of animal and time). Figure 2A shows that for the BM and PB transcript data, as well as the CBC data, approximately 30% of the variance is among animal and 10% among timepoints. For the metabolomes by contrast, only 15% of the variance is among animals with a slightly larger proportion due to timepoint.

**Table 1:** **Principle components of variation**.

**PC**	**PVE^a^**	**Animal^b^**	**Timepoint^b^**	**Sig. drug^c^ (effect)**
BM1	14.5%	0.67	0.18	3 × 10^−5^(Pre high)
BM2	10.2%	0.92	0.02	0.09^ns^
BM3	7.1%	0.15	0.74	2 × 10^−4^(inter low)
BM4	6.8%	0.91	0.05	0.0052 (pre high)
BM5	5.8%	0.80	0.13	0.011 (post low)
PB1	12.0%	0.94	0.02	0.59^ns^
PB2	8.2%	0.97	0.01	0.48^ns^
PB3	7.4%	0.87	0.01	0.75^ns^
PB4	7.0%	0.97	0.01	0.98^ns^
PB5	5.5%	0.12	0.14	0.36^ns^
AE1	17.3%	0.23	0.41	0.35^ns^
AE2	8.9%	0.13	0.47	0.14^ns^
AE3	7.3%	0.23	0.47	0.58^ns^
AE4	6.2%	0.41	0.34	0.13^ns^
AE5	4.9%	0.22	0.37	0.07^ns^
C18_1	18.5%	0.28	0.23	0.17^ns^
C18_2	9.3%	0.20	0.59	0.11^ns^
C18_3	6.6%	0.40	0.17	0.15^ns^
C18_4	6.3%	0.28	0.31	0.49^ns^
C18_5	4.8%	0.22	0.38	0.38^ns^

a *Amount of total variance explained by the PC*.

b *Amount of variance explained by Animal or Timepoint*.

c *Significance of Drug effect (pre vs. post vs. inter for transcriptome; post vs. inter for metabolome), also showing which effect was differentiated*.

The unexplained residual variance could be due to undefined biological sources, animal-by-timepoint interactions, random sampling variance, or technical error. To control for contributions of the latter, we reduced the datasets by removing the low-abundance features with the greatest coefficients of variation. Consistent with published findings (Rapaport et al., 2013), both RNA-Seq and MS have a strong relationship between abundance and variability, and based on the plots we adopted heuristic cutoffs of 5 log2 units for the transcripts and 17 log2 units for the metabolites. Figure 2B shows the variance components analysis based on the remaining features. In the PB, almost 70% of the variance is among animals, and in the BM approximately 50%. The temporal contribution drops to less than 5% for the PB, but increases to 20% for the BM. These results confirm that measurement error is a major contributor to estimation for low abundance transcripts with RNA-Seq. By contrast, the variance components for both metabolite columns is relatively unaffected by the data reduction, with both animal and time each continuing to explain approximately 15% of the overall variance. This may reflect filtration of the metabolites with the highest technical variance during peak calling.

### Hierarchical clustering of the omic data

**Figure 3:**
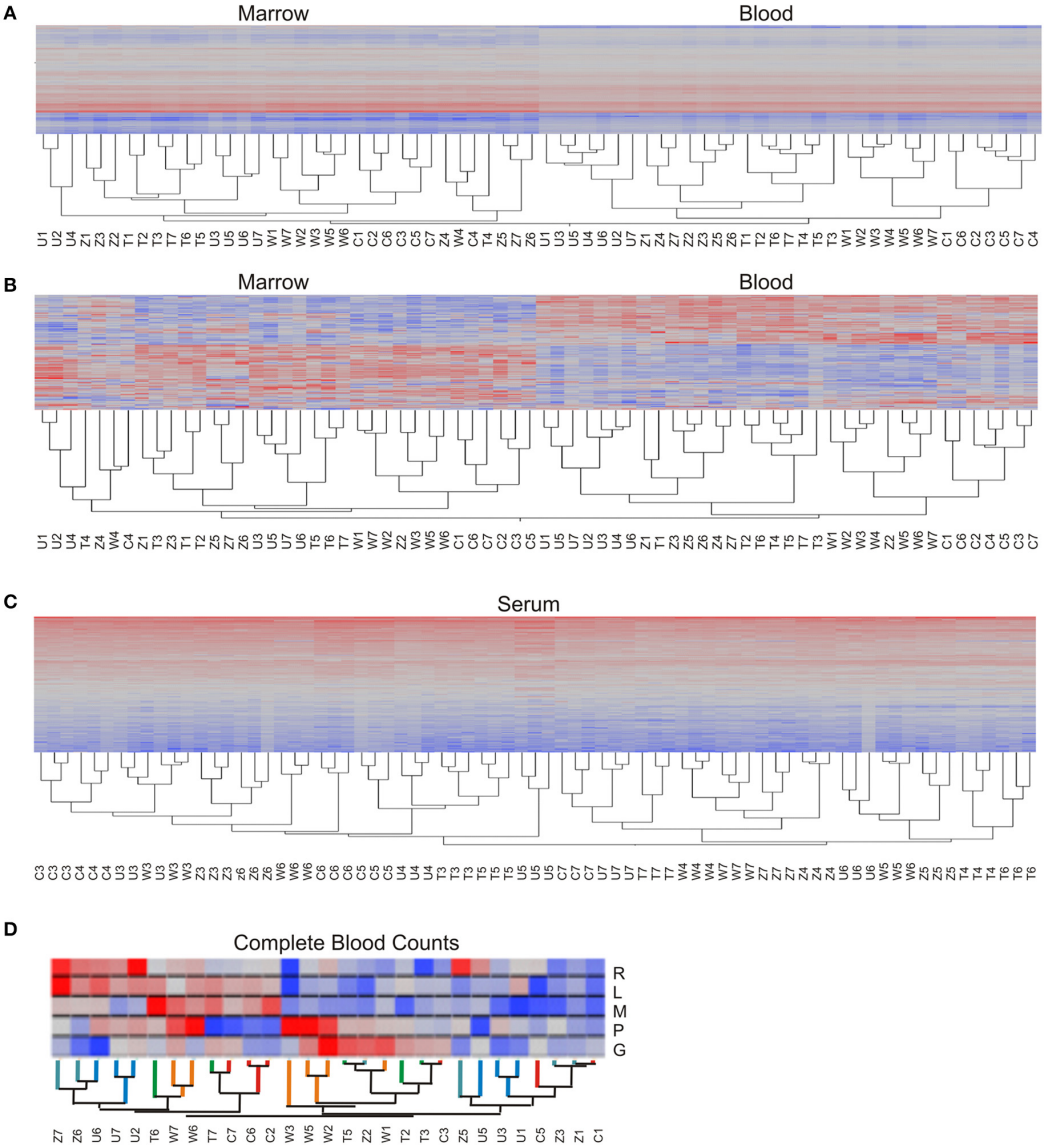
**Two-way hierarchical clustering**. Each heat map shows the abundance of each transcript or metabolite (rows) in each sample (column) with red indicating high expression, blue low, gray intermediate. **(A)** Transcriptome, based on absolute log-2 intensity estimates, and **(B)** based on standardized log-2 intensities, in both cases combining both BM and PB in the same clustering. **(C)** Plasma metabolome, where each column is a technical replicate, showing almost perfect alignment of each of the three replicates of each sample. **(D)** Complete blood counts are clustered with the branches of the dendrogram colored according to the identity of the animal, and cell types ordered as Red Blood Cells, Lymphocytes, Monocytes, Platelets, and Granulocytes (R, L, M, P, G respectively). Each macaque is abbreviated as C, T, U, W, or Z for RCs13, RTi13, RUn113, RWr13, or RZE13 respectively, and numbers refer to timepoints.

The preceding analysis tells us that both animal and time influence gene expression, but not which animals or timepoints are more similar. A quick means of visualizing these relationships is by two-way hierarchical clustering (Figure 3; Eisen et al., 1998). Applied to the raw data, in a joint analysis of the BM and PB, the gene expression of the two tissue types is clearly distinct, and the greater contribution of animal to the PB than the BM is seen by the perfect clustering of each of the seven timepoints within each animal grouping (Figure 3A). In the BM, there is some mixing of samples across animals, but it is also striking that TP4 is somewhat distinct since the data from four of the five animals cluster together. After standardization to *z*-scores for each gene, which removes the effect of overall abundance level for each transcript on the hierarchical clustering, these relationships are largely maintained (Figure 3B). The separation of TP4 in the BM is enhanced, although the clustering within animals is disrupted for a few PB samples. The situation for the metabolome is very different (Figure 3C) as neither the animals nor timepoints form discrete clusters. Both LC columns have almost identical topologies (data not shown), and technical variability does not explain these results since almost without exception all three technical replicates of each metabolite sample cluster adjacent to one another.

With respect to blood cell counts, the hierarchical clustering topology of monocytes, lymphocytes, granulocytes, RBC, and platelets did not correspond to either the transcriptome or metabolome topologies. The final two timepoints (TP6 and TP7) cluster to the exclusion of the earlier timepoints with a couple of exceptions, and within the two large clusters the individual animals are adjacent. However, there is no strong relationship between blood cell counts and overall gene expression (Figure 3D). Since each blood cell type has a characteristic expression profile which allows each cell to perform its specified role(s), including the limited mRNA complement in a nuclear RBC, we hypothesized that the macaques that clustered together in the expression profile would also have similar levels of the major cell types. However, we do not observe such a trend: macaques *RCs13* and *RWr13* (relative to *RTi13*, *RUn13*, and *RZe13*) form two sets of transcript profiles, whereas *RTi13* and *RWr13* are most similar for CBC with *RZe13* the most variable. Therefore, we conclude that the CBC is capturing information about the system that is non-redundant with the transcriptome. This result is particularly striking when considering that both the transcriptome datasets as well as the CBC datasets have the variance component of animal explaining more than 30% of the variance.

### Integration of the transcriptome and metabolome profiles

**Figure 4:**
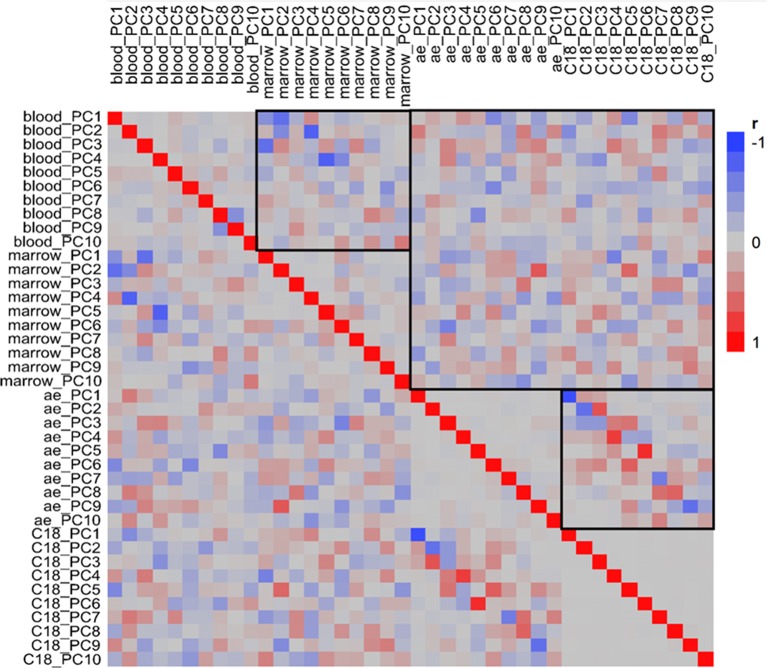
**Pairwise correlation of principal components**. Heatmap shows correlations of the first 10 principal components for each of the four omic datasets ordered by peripheral blood, bone marrow, AE and C18 (blue negative, red positive, stronger hues indicate stronger correlation). The highlighted boxes in the top left and bottom right quadrants show the correspondence between PC scores for the two transcriptomes and two metabolome columns, respectively; stronger colors along the diagonal indicate that those PC are capturing similar signals. Although there are scattered stronger colors in the top right quadrant comparing transcriptome and metabolome, the actual correlations are not significant.

These analyses suggest that the transcriptomes and metabolome are poorly correlated overall across all timepoints and animals, but do not exclude the possibility that subsets of features in the BM, the PB, or the plasma may be co-regulated. To test this using our top-down strategy, we evaluated the covariance between the major PC of each of the four omic datasets. Figure 4 shows the pairwise regression coefficients for each of the first 10 PC, allowing for the possibility that minor PC involving strong covariance of a small number of transcripts or metabolites may contribute.

The pattern that emerges is informative in many ways. Firstly, it shows that the two metabolomic datasets are highly correlated. This is to be expected since metabolites are being measured from the same plasma sample; the difference between the two datasets is the use of different liquid chromatography columns to optimize peak resolution across different classes of metabolites (broadly, sugars and amino acids on the AE anion exchange column, and lipids on the C18 column). PC1 and PC2 scores for the two columns are highly significantly correlated; many lower PC scores are also correlated. Unlike the metabolomic datasets, the two transcriptomic datasets, representing the BM and PB, do not show as much correlation (Figure 4, top left quadrant). Statistical analysis however does indicate that PC1, PC2, PC3, and PC4 in the PB are significantly correlated with PC2, PC4, PC1, and PC5 from the BM, respectively (Bonferroni corrected *p* < 0.05). Such a result is not unexpected considering that the two compartments have different functions yet one (blood) is composed of cell populations derived from the other (marrow). In some cases the sign of the regression is negative, but this is simply a function of PCA which commonly reverses signs and order of PC due to sampling variance. One difference between the compartments is that the marrow contains many cell types that are rapidly dividing whereas most of the cells in the blood are likely to be post-mitotic and terminally differentiated.

Strikingly, there is no significant correlation between the transcriptome PC scores and the metabolome PC scores across animals and timepoints. Figure 4 (top right box) seems to show some relationships, but none of these are significant after multiple testing correction. We also explored 2-block partial least square analysis (González et al., 2012) to identify minor variance components that may correlate in a joint analysis, but did not observe any significant enrichment between the two data types. This could be explained by the fact that the transcriptome of these two immune compartments is contained within the cell whereas the metabolome that we are interrogating is in the plasma. Furthermore, the plasma is not only influenced by metabolites from blood cells, but by metabolites secreted from all tissues in the body and taken up from the environment.

The correlation of the major PC between the PB and the BM datasets is dominated by among animal differences from the variance component analysis, but also includes a temporal component. The pre-drug samples in the BM are distinct from the post- and inter-drug samples, but TP4 is the most differentiated. In the PB, the baseline sample (TP1) is most differentiated, but TP4, and, even more strongly, TP5 are also somewhat divergent from the remaining samples. To assess the significance of the overlap, we extracted the genes that were up-regulated in the TP4 samples from the BM and performed a binomial sign-test of whether the same genes were up-regulated at TP5 in the PB. The result was highly significant (*p* < 3 × 10^−16^). A similar result was obtained for the down-regulated genes, but the control comparison of TP6 and TP7 in the PB for the same up- and down-regulated genes and did not result in any enrichment (*p* = 0.74). These data show that differential gene expression in the BM is reflected in the PB with a time lag (though contamination of BM with PB cannot be excluded). Note as well that in both tissues the TP4/5 differentially expressed genes are similar to the TP1 genes, but with opposite sign of effect, implying that genes up-regulated at baseline are down-regulated at TP4/5, and vice versa.

### Identification of drug-responsive genes and metabolites

The major temporal component of variation is not a cyclical response to drug administration, which would have produced a pattern where TP3, TP5, and TP7 were distinct from TP4 and TP6 and again from TP1 and TP2. None of the first 10 PC in PB showed a significant effect of drug administration on gene expression by analysis of variance with pre, inter and post levels of drug (Table 1). We nevertheless employed three strategies to identify potential drug-responsive genes: axis of variance analysis, pathway-oriented analysis, and gene set enrichment analysis.

**Figure 5:**
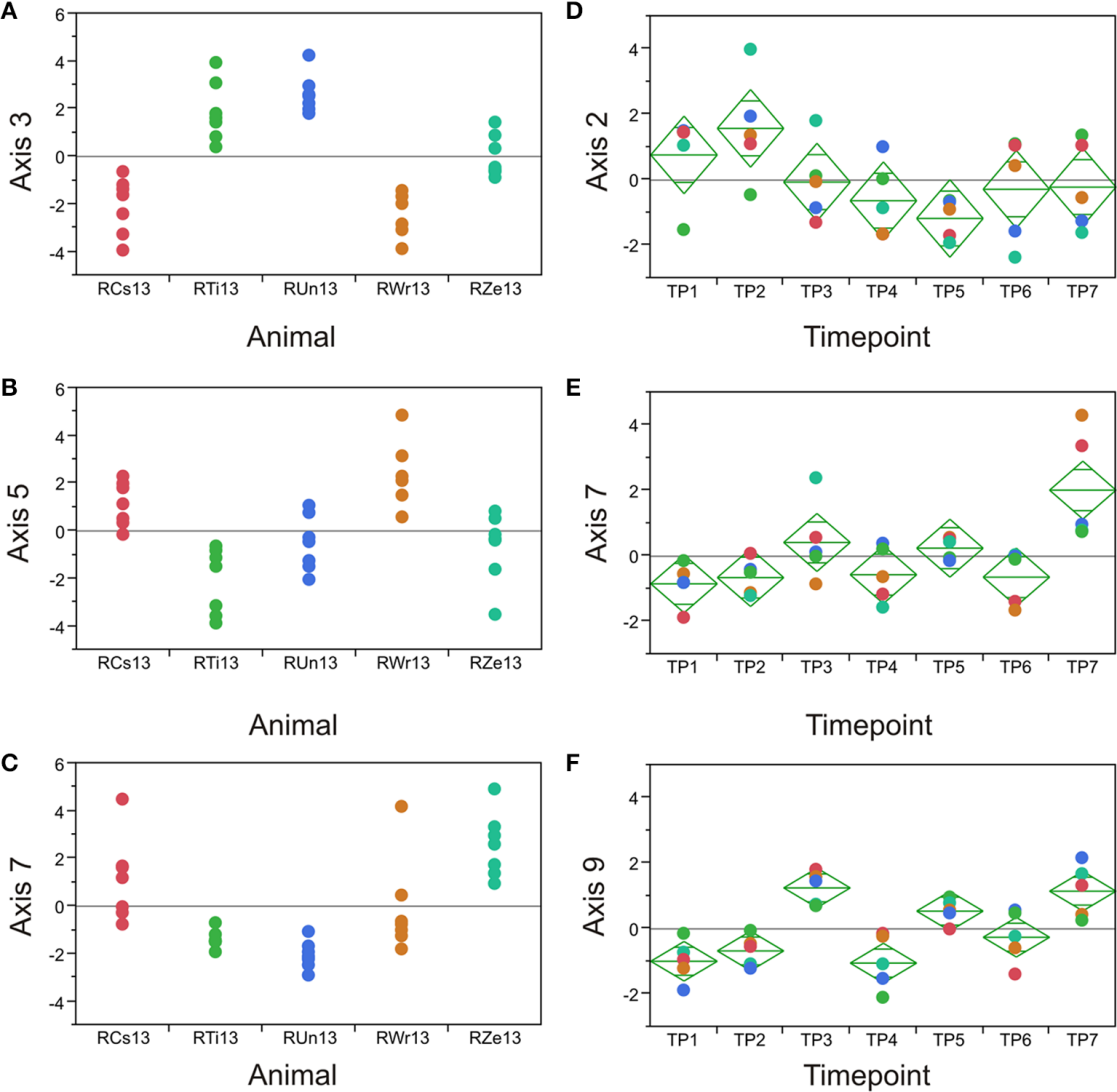
**Axis of variance analysis**. Each plot shows the indicated Axis score (PC1 of the 10 BIT for the Axis) in the five animals **(A–C)** or at the seven timepoints **(D–F)**. In bone marrow, Axes 7 and 9 are significantly differentiated at TP3, 5, and 7, the post-drug timepoints.

The requirement that PCs are orthogonal to one another introduces a statistical bias that is well-known to obscure underlying biology (Biswas et al., 2008). Consequently, we employed an alternate partitioning of the transcriptional variance based on conserved patterns of covariance of axes of gene expression that are observed in all large human PB transcriptome datasets (Preininger et al., 2013). Each of 9 axes is defined by 10 BITs that are highly co-regulated, and the first PC of these BITs is used as a measure of activity of genes in the axis. Each axis is thought to represent an aspect of immune function, such as T- or B-cell signaling (Axes 1 and 3), innate immune activation (Axis 5) or an interferon-related axis (Axis 7). We confirmed that the BIT are co-regulated in macaques, in both the PB and the BM, and asked whether they vary by animal or timepoint. Figure 5 shows highly significant among animal effects in the BM for Axes 3, 5, and 7. Importantly these data also capture a drug response effect that is not evident from the standard principal components, as Axes 7 and 9 are clearly differentially expressed at TP3, TP5, and TP7 in the BM, representing the post-drug samples. Axis 7 is also significantly divergent in the PB, as is Axis 3, while Axis 9 shows a non-significant trend (Table 2).

**Table 2:** **Axes of variance analysis**.

**Axis**	**Bone marrow**			**Peripheral blood**		
	**PVE by PC1^a^**	**Sig Animal^b^**	**Sig Drug^c^**	**PVE by PC1^a^**	**Sig Animal^b^**	**Sig Drug^c^**
1	45	0.0034	0.0006	43	ns	ns
2	77	8 × 10^−6^	0.0061	68	0.0002	0.0025^*^
3	63	1 × 10^−10^	0.0169^*^	91	2 × 10^−13^	ns
4	25	ns	ns	41	0.0005	ns
5	55	5 × 10^−6^	0.0022	73	0.0003	ns
6	35	0.0066	0.0005	29	0.0002	ns
7	54	3 × 10^−6^	0.0008^*^	76	3 × 10^−5^	0.0267^*^
8	45	ns	0.0183	58	2 × 10^−5^	ns
9	35	0.0002	1 × 10^−6^^*^	44	0.0234	ns

a *The percent of variation in the BIT explained by PC1 (>35% implies strong covariance)*.

b *The signficance of the among-animal effect*.

c *The significance of the pre-/post-/inter-drug treatment after removing the animal effect*.

* *Implies the post-drug treatment effect was extreme relative to pre- and inter-drug*.

Analysis of variance at the level of individual genes was also effective at recovering timepoint specific responses in the BM, but not initially in the PB: Table 3 lists the number of features significant at a False Discovery Rate of 5% (Benjamini and Hochberg, 1995). Recognizing that animal effects may obscure the temporal differences, we also ran the model with “animal” included as a random statistical effect, and recovered almost twice as many timepoint-responsive transcripts in the BM, and 292 in the PB. Similarly, ANOVA of the metabolome yielded many more significant timepoint-responsive metabolites after inclusion of “animal” as a random effect.

**Table 3:** **Differential gene expression between timepoints**.

**Data type**	**Tissue**	**Without animal in model**	**With animal in model**
RNA-Seq	Bone marrow	3678	6483
RNA-Seq	Whole blood	0	292
AE MS	Plasma	651	927
C18 MS	Plasma	1254	1992
CBC	Whole blood	10	13

*Table shows number of genes significant at 5% FDR rate for each data type, contrasting seven timepoints for RNA-Seq, and 5 timepoints for metabolomics*.

The most interesting timepoint effect with respect to drug exposure is where each of the post-drug timepoints is greater (or less) than the immediately preceding pre-drug timepoint, namely TP3 > TP2, TP5 > TP4, and TP7 > TP6. Again, this situation was only observed in the BM: 73 genes were consistently greater post-drug, and 25 consistently less strongly expressed post-drug, but no genes satisfied this criterion in PB (paired *t*-test, *p* < 0.05 at each of the three comparisons in 5 animals). A list is provided in the Supplementary data, and is notable for multiple immune-related genes, including TLR4, IL1RAP, IL1RAP, IL10RB, and MAP2K1.

### Pathway-oriented enrichment analyses

**Figure 6:**
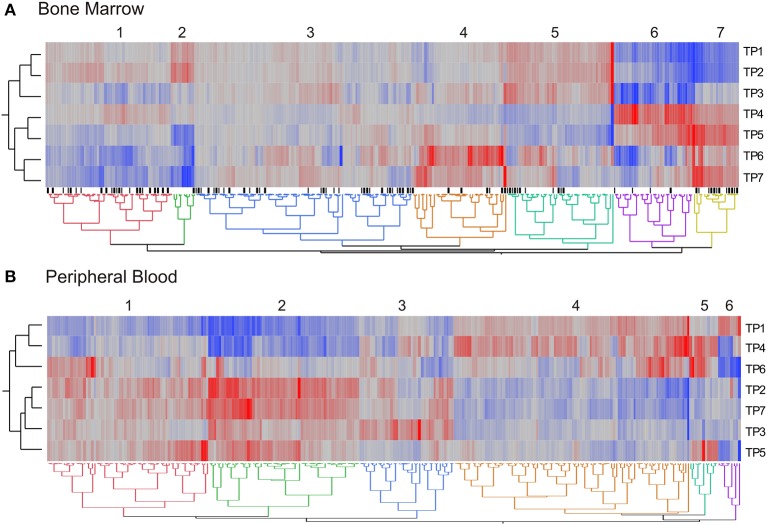
**Differential representation of pathways across timepoints**. The two-way hierarchical heat maps summarize co-expression of genes within pathways in the bone marrow **(A)** and peripheral blood **(B)**. Data points are the mean PC1 score for each of 270 KEGG pathways. Rows are timepoints, and the major clusters of PC1 scores are indicated. Black tick marks below the heatmap in **(A)** indicate pathways that are significantly different for the contrast of post- versus pre-drug treatment.

We next visualized the broad distribution of gene expression in pathways across the time course of pyrimethamine treatment by performing hierarchical clustering of a summary measure of each of 270 KEGG pathways. For each pathway with at least five transcripts expressed in both BM and PB, we generated the first principal component (PC1) of all of the transcripts annotated to the pathway, measured in all five monkeys at seven timepoints. These pathway PC1 values were averaged across the monkeys, and clustered by Ward's method in JMP. Figure 6 shows heat maps of the average PC1 scores for BM (A) and PB (B) with red corresponding to a high positive score, generally high transcript abundance, and blue a negative score, generally lower abundance on average.

In the BM, we observed seven clusters of pathway PC1 scores, with the major division of timepoints grouping TP1, TP2, and TP3 separately from TP4 through TP7. There was no clustering of pathways at the three post-drug timepoints (TP3, TP5, and TP7). In the PB, we observed just six clusters of pathway PC1 scores, with the major division of timepoints separating TP1, TP4, and TP6 from the remainder. Again, there was no evident clustering of the post-drug timepoints. The grouping of TP4 and TP6 corresponds to expected absence of drug, as does the baseline TP1, but this does not seem to relate to drug exposure since TP2 sampled immediately prior to the first drug administration, groups with the post-drug samples.

Comparing both sample types, 10% of the cluster identities in the PB are explained by the cluster identities in the BM (Pearson Chi-square, *p* < 10^−6^). However, this also means that the majority of the pathways change their average PC1 profile between the BM and the PB. There are nevertheless some interesting clusters. For example, the small green cluster 2 to the left in Figure 6A that is high prior to drug administration and low at the final three timepoints includes Ras and Rap 1 signaling, purine metabolism, and infectious disease response. By contrast, the yellow cluster 7 that is more highly expressed uniformly after first drug administration includes inflammatory autoimmune pathways, as well as extracellular matrix and cell adhesion. Most of the DNA repair and recombination pathways show the inverse pattern (clusters 4 and 5) implying down-regulation after persistent exposure to pyrimethamine, as might be expected due to reduction of cell division in response to folate inhibition. In the blood, the small blue-green cluster 5 that is high at TP4, TP5, and TP6 involves diverse pathways indicating perturbation of a variety of aspects of cellular physiology during that interval of time.

These trends were not necessarily consistent across all five monkeys. Similar hierarchical clustering of the 270 pathway PC1 scores of all 5 animals showed that two (RCs13 and RWr13) have quite similar profiles, while another two (RUn13 and RZe13) are only similar if TP4 is withdrawn from the analysis. Intriguingly, these pairs of monkeys were each housed together in the same cage, but there is no way of knowing whether that is coincidence or reflects an effect of shared environment. The result does however underline the conclusion that any effect of drug administration is to a large extent animal-specific.

### Targeted and gene set enrichment analysis

A disadvantage of the pathway-oriented approach is that it assumes that the covariance of genes within pathways that is captured by PC1 represents the most relevant aspect of perturbed gene expression. A more common approach is to identify differentially expressed genes and then ask whether they are enriched in particular pathways. We thus applied Gene Set Enrichment Analysis (GSEA) to the dataset, focusing on genes that are globally altered at the 5% FDR level following drug treatment, namely different between the two pre-drug samples and all five post- and inter-drug samples. In the BM, analysis of 4178 genes revealed 13 pathways down-regulated following pyrimethamine exposure, and 12 pathways up-regulated, at *p* < 0.001 and FDR *q* < 0.01; these are listed in Table 4. The down-regulated pathways reflect functions in the cell-cycle and metabolism including nucleotide biosynthesis and DNA repair, as well as oxidative phosphorylation (and glycolysis/gluconeogenesis trends in the same direction). The up-regulated pathways are all involved in immune signal transduction. Notably, in many cases the gene expression appears to be intermediate at TP3, suggesting a gradual transition in response to first drug administration that was reinforced with subsequent administrations and lasted several months.

**Table 4:** **Gene set enrichment analysis**.

**KEGG ID**	**Pathway name**	**Size**	***p***	**FDR *p***
**DOWN REGULATED AFTER PYRIMETHAMINE**				
3030	DNA replication	26	<0.001	<0.001
4110	Cell cycle	57	<0.001	<0.001
3410	Base excision rep.	18	0.002	<0.001
3420	Nucleotide excis'n	22	0.004	0.006
0072	Ox phosphoryl'n	45	<0.001	<0.001
0010	Glycolysis	23	0.011	0.019
0480	Glutathione	24	<0.001	<0.001
0240	Pyrimidine	37	<0.001	<0.001
0230	Purine	60	0.003	0.006
3040	Spliceosome	46	<0.001	<0.001
5016	Huntington's	56	<0.001	<0.001
5012	Parkinson's	44	<0.001	<0.001
5010	Alzheimer's	53	<0.001	<0.001
5322	SLE	34	0.005	0.008
**UP REGULATED AFTER PYRIMETHAMINE**				
4660	TCR signaling	44	<0.001	<0.001
4650	NK-mediated cytotox	34	<0.001	<0.001
4630	JAK-STAT signaling	36	<0.001	0.001
4070	PI signaling	19	<0.001	0.001
4210	Apoptosis	23	<0.001	0.011
4662	B cell receptor signaling	21	<0.001	0.014
4370	VEGF signaling	19	0.002	0.013
4150	mTOR signaling	19	0.002	0.015
4310	WNT signaling	42	0.007	0.022
4722	Neurotrophin signaling	40	0.007	0.025
4012	ErbB signaling	24	0.007	0.028
4514	Cell adhesion	37	0.007	0.031

**Figure 7:**
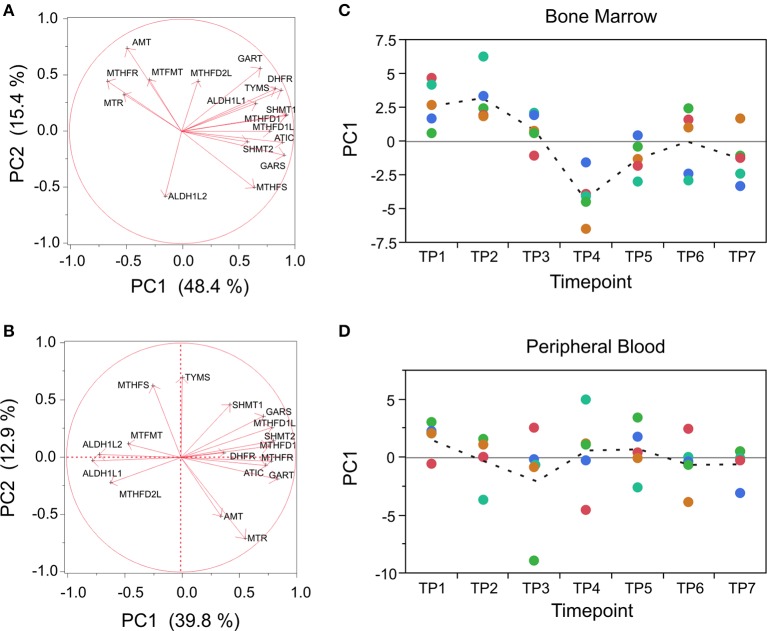
**Targeted analysis of the folate pathway. (A,B)** Loadings of the first two PC for each of 17 genes in KEGG map00670 (One carbon pool by folate) in BM and PB, also indicating the percent variation explained by each PC. Note that MTHFR switches direction effect between the cell sources. **(C,D)** Corresponding profiles across the timecourse, showing decline in PC1 generally in BM following drug exposure, but no significant differential expression in PB. Colors correspond to the five monkeys as in Figure 5.

A good example of this is provided by focused analysis of the one-carbon pool by folate pathway, which we expected to be influenced by pyrimethamine, since the drug functions by inhibiting the enzyme dihydrofolate reductase (DHFR). The pathway was too small to include in the GSEA, but nevertheless 12 of 17 genes expressed in the macaque and annotated to KEGG map00670 are positively co-regulated in both BM and PB samples, with PC1 capturing 51% and 40% of the variance, respectively (Figures 7A,B). The trajectory of this score trends downward beginning at TP3 in the BM and over half the variance is among timepoints (ANOVA *p* < 0.0001), whereas in the PB there is no differential expression (Figures 7C,D).

In the PB, purine (KEGG map00230) and pyrimidine (KEGG map00240) metabolism pathways both show a very large coordinated reduction in PC1 after TP1, namely before the first drug administration, and remain low throughout the experiment. Similarly, oxidative phosphorylation (KEGG map00190) is dominated by a transition that precedes drug administration, as is glycolysis (KEGG map00010), although it occurs in the opposite direction (i.e., gene expression increases). These results suggest that the animals experienced a shift in their major mode of energy production in the circulating blood cells after introduction into the experimental cages. Fatty acid biosynthesis also shows interesting patterns that we do not have space to describe in detail. All of these observations await confirmation at the metabolite level once the annotation of the m/z features on the platform is more advanced.

### Bayesian network analysis of the transcriptome

**Figure 8:**
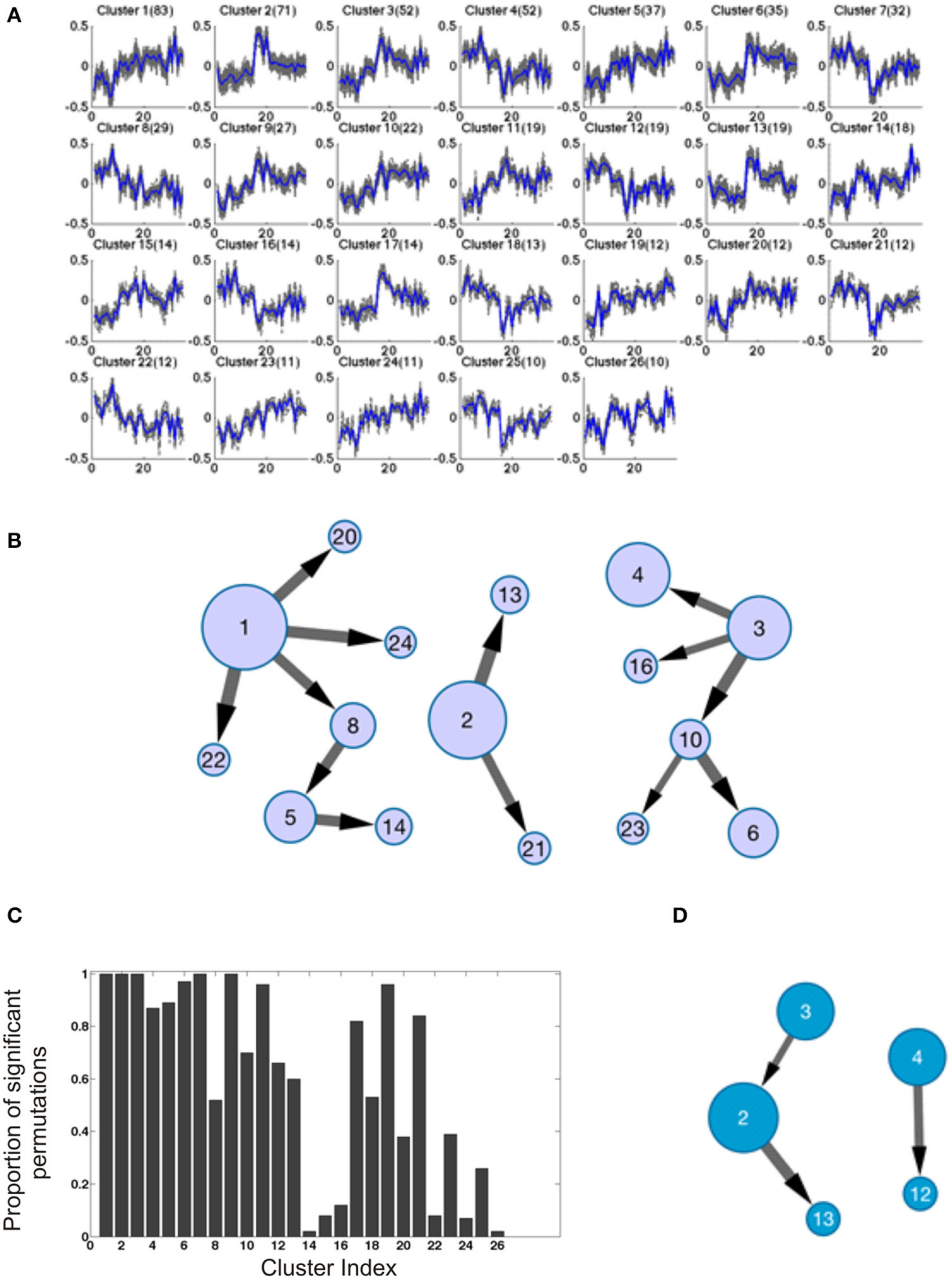
**Bayesian Network analysis. (A)** The results of quality-based clustering on BM transcriptional data provide tight clusters of coregulated genes used as input for Bayesian network inference. **(B)** The resulting robust network, defined as those connections present in at least 50% of all subsample analyses for each of four different permutations of the dataset. The size of nodes indicates the size of the clusters (also included in **A**), and the size of edges connecting nodes reflects the relative likelihood of a connection based on its overall frequency of occurrence across subsample replicates. **(C)** Statistical testing of the significance of correlations within clusters of PB data derived from BM data clustering. For each cluster, 100 random samples of genes of the same size of the cluster were compared to the PB data using the BM clustering of genes. The distributions of gene profile correlations to the centroid of their cluster were compared using a one-tailed Kolmogorov–Smirnov test. Histogram bars represent the number of random samplings showing statistically significant increases in correlation of the actual data compared to random data. **(D)** The PB network derived using BM clusters; of note there is one conserved connection between this and the BM network.

Finally, we adopted an orthogonal exploratory approach to describe networks of highly co-regulated genes. Each of the 1000 genes most differentially expressed relative to drug administration in the BM samples (that is, in the comparison of post- vs. inter-drug timepoints) were carried forward to quality-thresholded clustering (De Smet et al., 2002). We identified 26 clusters of 10 or more transcripts, the first 4 of which have at least 50 transcripts each (Figure 8A). Permutation of sample labels across timepoints or the entire data set never identified this degree of covariance: full permutation of sample labels for each gene independently recovered zero clusters, while permutation of timepoints within animals for each gene independently yielded just one cluster with two genes, indicating that the clusters found were not artifacts of the underlying data distributions while also increasing confidence that the clusters are biologically motivated. Permutation of animals within timepoints recovered a similar number and size of clusters as the true data, indicating that animal effects had been largely (but not completely) removed in generating the residuals. Since Bayesian network inference is typically much more robust and reliable with many more than the 35 samples available in this study, we increased the effective number of observations per variable by treating each gene (at each timepoint) as an observation of the behavior if its cluster. To do this, we concatenated the top 10 genes most closely correlated with the cluster centroid, yielding 350 observations for each of the 26 clusters. The most robust and likely connections in the emergent networks were then determined by subsampling and permutation as described in the Methods.

An efficient and powerful method for Bayesian network structure learning, the Sparse Candidate Algorithm (Friedman et al., 1999), was used to uncover the potential connections between the clusters. Networks were inferred for 1000 randomly generated subsamples of 90% of the data for each gene to ensure the robustness of the learning results; all connections shown in Figure 8B satisfy the criterion that each connection must exist in at least 50% of all of the resampling simulations for the original data, and in each of the three permutations of that data; we found an average overlap of 66.7% of interactions conserved between the original dataset and each of the three shuffles for the BM data, with 13 connections consistently detected in all datasets (see detailed descriptions of robustness testing in the Methods). Core features of this BM network were further investigated by inspection, and validated by gene set enrichment analysis (Subramanian et al., 2005). For instance, cluster 1 shows complimentary patterns to clusters 8 and 22, while it is most similar to clusters 20 and 24. The core genes in each of these clusters suggest functions in immune T-cell responses. Clusters 1 and 3 are “hubs” with a relatively high degree of connectivity in a graph that is otherwise quite sparse.

Although there was little evidence for significant differential expression among the three drug response classes (pre-, post-, and inter-) in the PB, we nevertheless assessed whether the BM cluster modularity may be present in the PB. Projected onto the PB, many of the BM clusters appeared to be co-regulated. To statistically validate this inference, for each cluster we computed the correlation of each gene with the centroid in the PB data, and compared the observed distribution with that of 100 random samples of the same size as that cluster, taken from the 660 transcripts contained in all of the clusters. Figure 8C shows the proportion of permutations showing a significant deviation in the direction of stronger concordance in the observed data, providing good evidence for cluster conservation in the PB for 12 of the 13 largest clusters, as well as several of the smaller ones. Furthermore, the Bayesian network approach identified a number of robust connections in the PB data, showing an average 37.5% overlap between the original data and any of the permutations, and three connections were observed in common across all permutations (Figure 8D). One of those three connections was observed in both the PB and the BM, implying robust dependence of cluster 13 on cluster 2 between the two compartments. Of note, the genes in both of these clusters have been implicated in 6 and 24 h responses to the anti-tumor aminopeptidase inhibitor Tosedostat (Krige et al., 2008). The other two connections (cluster 3 to 2, and cluster 4 to 12) are found in PB but not in BM, suggesting that not only is there conservation of modularity between the compartments, but that new relationships using the modularity of one compartment can be observed in the other.

**Figure 9:**
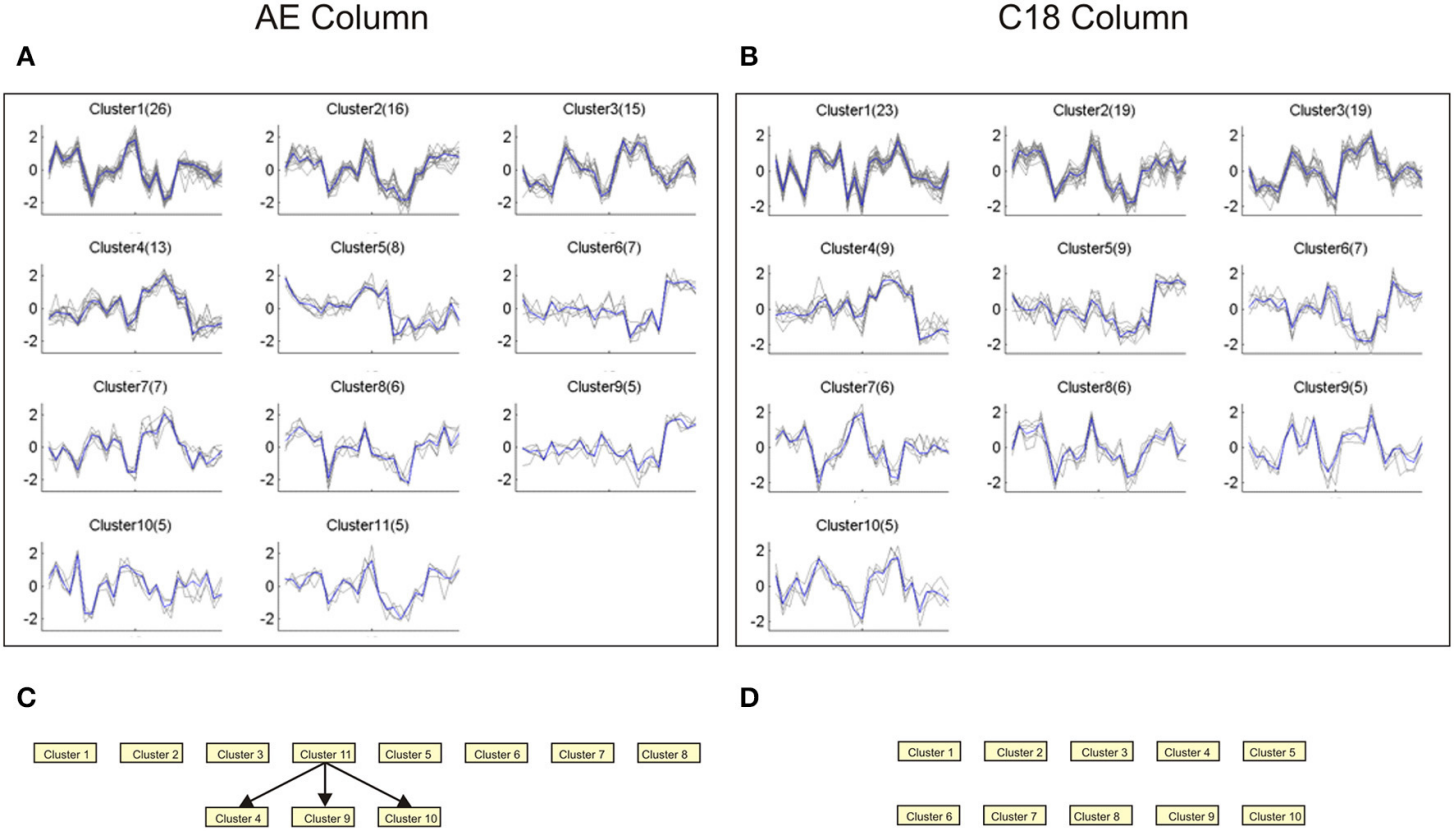
**Quality-thresholded clustering of metabolomics data**. Each plot shows the standardized levels of the indicated number in parentheses of m/z features in Qt-clusters that have at least 5 features. The order of samples along the x-axis is {RCs18, RTi18, RUn18, RWr18, and RZe18} at TP3, 4, 5, 6, and 7, and the solid blue line indicates the centroid of the cluster. **(A)** AE column. **(B)** C18 column. **(C,D)** Bayesian networks assembled on clusters with five metabolites and seven levels of discretization, and 80% subsampling threshold, similar to the analyses used to generate the transcriptomic networks in Figure 8B.

Application of a similar pipeline to the metabolome data also revealed novel structure to the data. Since there are fewer differentially abundant m/z features in the plasma, this analysis was performed on 500 features for each column with relatively high false discovery rates on the post- vs. inter-drug samples, namely 28% for C18 (*p* < 0.01) and 36% for AE (*p* < 0.031). Quality-thresholded clustering identified 11 and 10 clusters respectively with more than 5 m/z features, and 4 and 3 with more than 10 m/z features. The profiles of the larger clusters in each of the two columns are concordant (Figures 9A,B), and most were also recovered in a joint analysis of both columns, with approximately double the number of features. This suggests that the two columns reproduce the same tight clusters of metabolites across animals and times, although the actual m/z values do not match, suggesting that different ionizations or adducts may have been included in the selected features for each column. After Bayesian Network analysis, three robust connections were observed between AE clusters, but none with the C18 data (Figures 9C,D).

To investigate possible integration of the metabolic and transcriptional data types, we first simply performed correlation analysis between the centroids of all of the clusters. The strongest interaction that was identified had a correlation coefficient of −0.61. Using Bayesian network inference on all clusters of size greater than 10 between BM transcriptional data and AE column metabolomics data (using only timepoints 3–7 for all based on availability of metabolomics data), we found little in the way of robust connections between the two data types (data not shown). There were no connections conserved across 50% of the subsampling analyses in each of the original and four permuted datasets; however, relaxing this criterion slightly to include any connection present for 50% of all subsampling runs across all four datasets (not 50% in each individual dataset) revealed one potential connection between the two data types.

## Discussion

This study with naïve healthy rhesus macaques precedes others that will involve specific infection and treatment regimens, and, importantly, it has served to establish logistics and methodologies for systems biological approaches requiring the monitoring and evaluation of clinical data, collection of PB and BM samples, and the integrative analysis of multiple omics and other datasets. The biological objective of this study was to use the combined power of transcriptomic and metabolomic profiling to investigate the effects of an anti-malarial drug on the physiology of the PB and BM. Five rhesus macaques were injected with a preparation of uninfected *Anopheles dirus* salivary glands, to mimic an inoculation of *Plasmodium* sporozoites, and then followed for 100 days with intermittent administration of pyrimethamine, a drug known to have an effect on the BM.

Ideally the unbiased top-down analytical approach that we adopted would identify components of variation in both the transcriptional and metabolomic domains that covary with drug administration, and enrichment analysis of both would point to a common aspect of metabolic regulation such as nucleotide biosynthesis. To some extent we were thwarted in this objective by three findings: (i) there is very low correspondence between the transcriptome and metabolome and no major components of variation correlate with repeated pyrimethamine administration; (ii) among animal effects dominate the transcriptome raising the possibility that pyrimethamine responses are variable among individuals and obscure any common response; and (iii) although the metabolomics platform reports thousands of features, annotation is not yet robust enough to support global enrichment analysis in this dataset (but see Li et al., 2013, for encouraging developments).

Additionally, we must acknowledge that this is a relatively small study, with just five monkeys and seven timepoints. The failure to detect strong drug responses or covariance of the blood and transcriptome may simply be a function of lack of statistical power. For example, although we can attribute the largest PC to specific sources of variance, those explaining much less than 10% of the variance might be regarded as noise, and are unlikely to replicate. That is one reason why we turned to the Axis of Variation analysis, since the axes have a more biological basis that is not as dependent on sample size. The pathway-oriented analysis also highlights how interpretation of single gene effects must be placed within the context of the major sources of variance, in this case animal effects and some temporal shifts that may not relate to drug administration. It is likely that much larger studies would be required to detect strong transcriptome-metabolome covariance: for example, our analysis of 20 strains of Drosophila profiled on four diets with considerable technical replication did suggest some specific examples of covariance despite general absence of correspondence of the major PC axes, and even in the presence of large genotype-by-diet interactions (Reed et al., 2014). Studies with hundreds of NHPs are impractical, so we must make do with analytical methods such as those reported here, but recognizing that there is low power and the potential to over-interpret those associations that are detected.

Nevertheless, several key findings contradict our expectations and highlight aspects of the biology that emerge from multi-omic analyses. Most striking is the magnitude of the among-animal differentiation of the transcriptome. This is even stronger in the PB than the BM, with almost perfect clustering of all seven timepoint samples within animals. Each of the major PC and Axes of variation are significantly different among animals. In the BM, an unknown variable caused TP4 to generate a markedly different profile common to all five macaques, yet the individual profiles return to the animal-specific baseline within weeks. Persistent among-individual differential expression in the PB has also been reported in humans (Whitney et al., 2003; GG unpublished), but here we demonstrate for the first time that the differential expression is initiated in the BM and the data suggests that it precedes and is independent of individual-specific environmental influences faced in the PB. Persistent inter-individual variation is less marked in the metabolome, but nevertheless present as previously observed by Park et al. (2009) in a human dietary intervention study.

The temporal component of transcriptional variance shows some sign of cycling with drug administration, but it is dominated by timepoints that are not primarily associated with recent administration of the drug. In the PB, TP1 is most divergent, possibly indicating incomplete acclimatization of the animals to their new experimental housing and experimental procedures. Indeed, one of the pathways elevated at TP1 denotes a generalized stress response, as we have previously observed in captive relative to free range red wolves (Kennerly et al., 2008). This effect is much less in the BM, and since TP1 and TP2 differ from the remaining timepoints for 2 of the first 5 PC and 3 of 9 Axes (e.g., Figures 5D,F). Consistent with the observation that pyrimethamine has an effect on the BM (Wickramasinghe and Litwinczuk, 1981) our data indicates that in the BM there is a global impact of pyrimethamine that persists throughout the experiment following the first administration. Then at TP4 in the BM and TP4 and especially TP5 in the PB, there is further differentiation of gene expression consistent with a heightened response to the drug. TP4 is an inter-treatment timepoint, over 20 days after the previous administration at a time when pyrimethamine should no longer be in circulation based on its half-life of 140 h (Almond et al., 2000). Based on this figure there should nevertheless be around one third of the administered dose still available at the post-drug timepoints (TP3, 5, and 7), but we do not know to what extent it would be directly available to cells in the BM or circulating in the blood. Consequently, it is possible that the relatively weak drug effects are because the animals are no longer functionally exposed to pyrimethamine at the sampled timepoints. We have been unable to correlate the change at TP4 with any variable such as a change in handler or cage conditions. The null hypothesis of no differential expression across time is rejected, but we do not have a clear alternate hypothesis for the effect.

In the metabolome, there is very good correspondence between the PC and the hierarchical cluster profiles of the two columns, but the major variance components do not correlate with either animal or drug response. Since retention times differ between the columns, and m/z peaks included in feature selection may be from different adducts for several metabolites, it is not straightforward to combine the analysis of both columns. The major temporal effect is at TP7, which shows a correlated response across all five animals. It is unclear whether this represents a long-term effect of more than two months of drug treatment, or some other unidentified stimulus, but it has no correlate in the transcriptome. Several hundred metabolite features are globally different in the post-drug samples, even though the major PC also differentiate the post- and inter-drug timepoints.

The antimalarial drug pyrimethamine interferes with folate by inhibiting the enzyme dihydrofolate reductase and disrupts the parasite life cycle by interfering with nucleotide metabolism and replication. It also affects host metabolism, and in fact folate supplementation is often used to sustain healthy erythropoiesis in pregnant women and infants (Titaley et al., 2010). Gene set enrichment analysis (Subramanian et al., 2005) of the transcriptome provides some evidence for effects on metabolism and cell division. Briefly, contrasting the pre- with the post- and inter-drug timepoints, some common pathways between the BM and PB, as well as BM-specific changes, are observed. The former are of a metabolic nature, including oxidative phosphorylation, pentose phosphate, glyoxylate, butanoate, and linoleic acid metabolism; the latter include multiple KEGG pathways related to the cell cycle such as DNA replication, recombination, and repair. Our Bayesian network approach also focuses attention on regulation of cell division since one of the key enrichments in the BM is with targets of Tosedostat, an anti-cancer drug that antagonizes aminopeptidase activity (DiNardo and Cortes, 2014). Results such as this generate hypotheses that can be tested by targeted metabolomics and manipulation of gene expression, suggesting a new integrative genomics approach to pharmacogenetics.

Our top down analyses also provide some important lessons regarding the joint use of different data integration strategies in MaHPIC (or similar) experiments where a relatively small number of individuals will be followed longitudinally during an intervention. While the principal components approach efficiently defines the major sources of variation, it misses important biological results and is not obviously the best strategy for integration of multiple omic and immunological measures. In particular, the axis of variation analysis picks up effects of drug administration on broad aspects of immune function, most notably interferon-related gene activity highlighted by Axis 7 in both PB and BM samples. It is unlikely in this case that the elevation of this axis is due to viral activity, but this result and weaker evidence for dysregulation of Axes 2 and 9 in the week after pyrimethamine administration show that the network of immune interactions is perturbed and that drug activity is not narrowly restricted to the immediate effects of folate. Finally, given the small number of animals and timepoints in this experiment, statistical power is low for formal hypothesis testing, but we begin to show how Bayesian Network analysis can tease out interaction effects that are not evident in univariate analysis or in analyses designed to capture the largest overall components of variance. The two immune compartments share clusters of co-regulated gene modules, but the connectivity of these differs between BM and PB samples. *Plasmodium* infection will have a much larger impact on the animals' physiology than the mock-inoculations described here, providing ample opportunity for exploring network-based modeling of the host-parasite interactions that underlie malaria infections, immunity, pathogenesis, and severe disease.

### Conflict of interest statement

The authors declare that the research was conducted in the absence of any commercial or financial relationships that could be construed as a potential conflict of interest.

## References

[B1] Aliferis C. Tsamardinos I. Statnikov A. (2003). Causal Explorer: a probabilistic Network Learning Toolkit for Biomedical Discovery. METMBS. Available online at: http://www.dsl-lab.org/ml_tutorial/Publications/Causal_Explorer.pdf

[B2] Almond D. S. Szwandt I. S. Edwards G. Lee M. G. Winstanley P. A. (2000). Disposition of intravenous pyrimethamine in healthy volunteers. Antimicrob. Agents Chemother. 44, 1691–1693. 10.1128/AAC.44.6.1691-1693.2000 10817730 PMC89934

[B3] Anders S. Huber W. (2010). Differential expression analysis for sequence count data. Genome Biol. 11:R106. 10.1186/gb-2010-11-10-r106 20979621 PMC3218662

[B4] Anders S. Pyl P. T. Huber W. (2014). HTSeq - a Python framework to work with high-throughput sequencing data. bioRxiv Available online at: http://biorxiv.org/content/biorxiv/early/2014/08/19/002824.full.pdf 10.1101/002824 PMC4287950 25260700

[B5] Bang J. W. Crockford D. J. Holmes E. Pazos F. Sternberg M. J. Muggleton S. H.. (2008). Integrative top-down system metabolic modeling in experimental disease states via data-driven Bayesian methods. J. Proteome Res. 7, 497–503. 10.1021/pr070350l 18179164

[B6] Benjamini Y. Hochberg Y. (1995). Controlling the false discovery rate: a practical and powerful approach to multiple testing. J. R. Stat. Soc. B 57, 289–300.

[B7] Biswas S. Storey J. D. Akey J. M. (2008). Mapping gene expression quantitative trait loci by singular value decomposition and independent component analysis. BMC Bioinformatics 9:244. 10.1186/1471-2105-9-244 18492285 PMC2424053

[B8] Boedigheimer M. J. Wolfinger R. D. Bass M. B. Bushel P. R. Chou J. W. Cooper M.. (2008). Sources of variation in baseline gene expression levels from toxicogenomics study control animals across multiple laboratories. BMC Genomics 9:285. 10.1186/1471-2164-9-285 18549499 PMC2453529

[B9] Bumgarner R. E. Yeung K. Y. (2009). Methods for the inference of biological pathways and networks. Methods Mol. Biol. 541, 225–245. 10.1007/978-1-59745-243-4_11 19381545

[B10] De Smet F. Mathys J. Marchal K. Thijs G. De Moor B. Moreau Y. (2002). Adaptive quality-based clustering of gene expression profiles. Bioinformatics 18, 735–746. 10.1093/bioinformatics/18.5.735 12050070

[B11] Devonshire A. S. Elaswarapu R. Foy C. A. (2010). Evaluation of external RNA controls for the standardisation of gene expression biomarker measurements. BMC Genomics 11:662. 10.1186/1471-2164-11-662 21106083 PMC3091780

[B12] Deye G. A. Gettayacamin M. Hansukjariya P. Im-erbsin R. Sattabongkot J. Rothstein Y.. (2012). Use of a rhesus Plasmodium cynomolgi model to screen for anti-hypnozoite activity of pharmaceutical substances. Am. J. Trop. Med. Hyg. 86, 931–935. 10.4269/ajtmh.2012.11-0552 22665596 PMC3366535

[B13] DiNardo C. D. Cortes J. E. (2014). Tosedostat for the treatment of relapsed and refractory acute myeloid leukemia. Expert Opin. Investig. Drugs 23, 265–272. 10.1517/13543784.2014.864276 24313331

[B14] Eisen M. B. Spellman P. T. Brown P. O. Botstein D. (1998). Cluster analysis and display of genome-wide expression patterns. Proc. Natl. Acad. Sci. U.S.A. 95, 14863–14868. 10.1073/pnas.95.25.14863 9843981 PMC24541

[B15] Frevert U. Nacer A. (2013). Immunobiology of *Plasmodium* in liver and brain. Parasite Immunol. 35, 267–282. 10.1111/pim.12039 23631610

[B30] Friedman N. Nachman I. Peer D. (1999). Learning Bayesian network structure from massive datasets: The “sparse candidate” algorithm, in Proceedings of the Fifteenth Conference on Uncertainty in Artificial Intelligence (UAI-99) (San Francisco, CA: Morgan Kaufmann Publishers Inc.), 206–215.

[B16] Galinski M. R. Meyer E. V. Barnwell J. W. (2013). *Plasmodium vivax*: modern strategies to study a persistent parasite's life cycle. Adv. Parasitol. 81, 1–26. 10.1016/B978-0-12-407826-0.00001-1 23384620

[B17] Giuliani A. Filippi S. Bertolaso M. (2014). Why network approach can promote a new way of thinking in biology. Front. Genet. 5:83. 10.3389/fgene.2014.00083 24782892 PMC3986556

[B18] González I. Cao K. A. Davis M. J. Déjean S. (2012). Visualising associations between paired “omics” data sets. BioData Min. 5:19. 10.1186/1756-0381-5-19 23148523 PMC3630015

[B19] Hafalla J. C. Silvie O. Matuschewski K. (2011). Cell biology and immunology of malaria. Immunol. Rev. 240, 297–316. 10.1111/j.1600-065X.2010.00988.x 21349101

[B20] Hartemink A. J. (2001). Discretization of genomic expression data, in Principled Computational Methods for Validation and Discovery of Genetic Regulatory Networks. PhD Thesis, Massachussets Institute of Technology.

[B21] Heyer L. J. Kruglyak S. Yooseph S. (1999). Exploring expression data: identification and analysis of co-expressed genes. Genome Res. 9, 1106–1115. 10.1101/gr.9.11.1106 10568750 PMC310826

[B22] Jones D. P. Park Y. Ziegler T. R. (2012). Nutritional metabolomics: progress in addressing complexity in diet and health. Annu. Rev. Nutr. 32, 183–202. 10.1146/annurev-nutr-072610-145159 22540256 PMC4031100

[B23] Kanehisa M. Goto S. (2000). KEGG: kyoto encyclopedia of genes and genomes. Nucleic Acids Res. 28, 27–30. 10.1093/nar/28.1.27 10592173 PMC102409

[B24] Kanehisa M. Goto S. Sato Y. Kawashima M. Furumichi M. Tanabe M. (2014). Data, information, knowledge and principle: back to metabolism in KEGG. Nucleic Acids Res. 42, D199–D205. 10.1093/nar/gkt1076 24214961 PMC3965122

[B25] Kennedy M. Fishbaugher M. E. Vaughan A. M. Patrapuvich R. Boonhok R. Yimamnuaychok N.. (2012). A rapid and scalable density gradient purification method for *Plasmodium* sporozoites. Malar. J. 11:421. 10.1186/1475-2875-11-421 23244590 PMC3543293

[B26] Kennerly E. Ballmann A. Martin S. Wolfinger R. Gregory S. Stoskopf M.. (2008). A gene expression signature of confinement in peripheral blood of red wolves (*Canis rufus*). Mol. Ecol. 17, 2782–2791. 10.1111/j.1365-294X.2008.03775.x 18466232

[B27] Kim D. Pertea G. Trapnell C. Pimentel H. Kelley R. Salzberg S. L. (2013). TopHat2: accurate alignment of transcriptomes in the presence of insertions, deletions and gene fusions. Genome Biol. 14:R36. 10.1186/gb-2013-14-4-r36 23618408 PMC4053844

[B28] Krige D. Needham L. A. Bawden L. J. Flores N. Farmer H. Miles L. E.. (2008). CHR-2797: an antiproliferative aminopeptidase inhibitor that leads to amino acid deprivation in human leukemic cells. Cancer Res. 68, 6669–6679. 10.1158/0008-5472.CAN-07-6627 18701491

[B29] Li S. Park Y. Duraisingham S. Strobel F. H. Khan N. Soltow Q. A.. (2013). Predicting network activity from high throughput metabolomics. PLoS Comput. Biol. 9:e1003123. 10.1371/journal.pcbi.1003123 23861661 PMC3701697

[B31] Moreno A. Cabrera-Mora M. Garcia A. Orkin J. Strobert E. Barnwell J. W.. (2013). *Plasmodium coatneyi* in rhesus macaques replicates the multisystemic dysfunction of severe malaria in humans. Infect. Immun. 81, 1889–1904. 10.1128/IAI.00027-13 23509137 PMC3676004

[B32] Park Y. Kim S. B. Wang B. Blanco R. A. Le N. A. Wu S.. (2009). Individual variation in macronutrient regulation measured by proton magnetic resonance spectroscopy of human plasma. Am. J. Physiol. Regul. Integr. Comp. Physiol. 297, R202–R209. 10.1152/ajpregu.90757.2008 19458279 PMC2711699

[B33] Pei B. Shin D. G. (2012). Reconstruction of biological networks by incorporating prior knowledge into Bayesian network models. J. Comput. Biol. 19, 1324–1334. 10.1089/cmb.2011.0194 23210479 PMC3513982

[B34] Preininger M. Arafat D. Kim J. Nath A. P. Idaghdour Y. Brigham K. L.. (2013). Blood-informative transcripts define nine common axes of peripheral blood gene expression. PLoS Genet. 9:e1003362. 10.1371/journal.pgen.1003362 23516379 PMC3597511

[B35] Rapaport F. Khanin R. Liang Y. Pirun M. Krek A. Zumbo P.. (2013). Comprehensive evaluation of differential gene expression analysis methods for rna-seq data. Genome Biol. 14:R95. 10.1186/gb-2013-14-9-r95 24020486 PMC4054597

[B36] Reed L. K. Lee K. Zhang Z. Rashid L. Poe A. Hsieh B.. (2014). Systems genomics of metabolic phenotypes in wild-type *Drosophila melanogaster*. Genetics 197, 781–793. 10.1534/genetics.114.163857 24671769 PMC4063932

[B37] Schroeder A. Mueller O. Stocker S. Salowsky R. Leiber M. Gassmann M.. (2006). The RIN: an RNA integrity number for assigning integrity values to RNA measurements. BMC Mol. Biol. 7:3. 10.1186/1471-2199-7-3 16448564 PMC1413964

[B38] Schwenk R. J. Richie T. L. (2011). Protective immunity to pre-erythrocytic stage malaria. Trends Parasitol. 27, 306–314. 10.1016/j.pt.2011.02.002 21435951

[B39] Soltow Q. A. Strobel F. H. Mansfield K. G. Wachtman L. Park Y. Jones D. P. (2013). High-performance metabolic profiling with dual chromatography-Fourier-transform mass spectrometry (DC-FTMS) for study of the exposome. Metabolomics 9, S132–S143 10.1007/s11306-011-0332-1 PMC4517297 26229523

[B40] Soneson C. Delorenzi M. (2013). A comparison of methods for differential expression analysis of rna-seq data. BMC Bioinformatics 14:91. 10.1186/1471-2105-14-91 23497356 PMC3608160

[B41] Stanisic D. I. Barry A. E. Good M. F. (2013). Escaping the immune system: how the malaria parasite makes vaccine development a challenge. Trends Parasitol. 29, 612–622. 10.1016/j.pt.2013.10.001 24176554

[B42] Subramanian A. Tamayo P. Mootha V. K. Mukherjee S. Ebert B. L. Gillette M. A.. (2005). Gene set enrichment analysis: a knowledge-based approach for interpreting genome-wide expression profiles. Proc. Natl. Acad. Sci. U.S.A. 102, 15545–15550. 10.1073/pnas.0506580102 16199517 PMC1239896

[B43] Tachibana S. Sullivan S. A. Kawai S. Nakamura S. Kim H. R. Goto N.. (2012). *Plasmodium cynomolgi* genome sequences provide insight into Plasmodium vivax and the monkey malaria clade. Nat. Genet. 44, 1051–1055. 10.1038/ng.2375 22863735 PMC3759362

[B44] Titaley C. R. Dibley M. J. Roberts C. L. Agho K. (2010). Combined iron/folic acid supplements and malaria prophylaxis reduce neonatal mortality in 19 sub-Saharan African countries. Am. J. Clin. Nutr. 92, 235–243. 10.3945/ajcn.2009.29093 20504976

[B45] Trapnell C. Roberts A. Goff L. Pertea G. Kim D. Kelley D. R.. (2012). Differential gene and transcript expression analysis of RNA-seq experiments with TopHat and Cufflinks. Nat. Protoc. 7, 562–578. 10.1038/nprot.2012.016 22383036 PMC3334321

[B46] Uppal K. Soltow Q. A. Strobel F. H. Pittard W. S. Gernert K. M. Yu T.. (2013). xMSanalyzer: automated pipeline for improved feature detection and downstream analysis of large-scale, non-targeted metabolomics data. BMC Bioinformatics 14:15. 10.1186/1471-2105-14-15 23323971 PMC3562220

[B47] Wang L. Wang S. Li W. (2012). RSeQC: quality control of RNA-seq experiments. Bioinformatics 28, 2184–2185. 10.1093/bioinformatics/bts356 22743226

[B48] Whitney A. R. Diehn M. Popper S. J. Alizadeh A. A. Boldrick J. C. Relman D. A.. (2003). Individuality and variation in gene expression patterns in human blood. Proc. Natl. Acad. Sci. U.S.A. 100, 1896–1901. 10.1073/pnas.252784499 12578971 PMC149930

[B49] WHO World Malaria Report. (2013). Available online at: http://www.who.int/malaria/publications/world_malaria_report_2013/en/

[B50] Wickramasinghe S. N. Litwinczuk R. A. (1981). Effects of low concentrations of pyrimethamine on human bone marrow cells *in vitro*: possible implications for malaria prophylaxis. J. Trop. Med. Hyg. 84, 233–238. 7321070

[B51] Wilhelm B. T. Landry J. R. (2009). RNA-Seq-quantitative measurement of expression through massively parallel RNA-sequencing. Methods 48, 249–257. 10.1016/j.ymeth.2009.03.016 19336255

[B52] Wolfinger R. D. Gibson G. Wolfinger E. D. Bennett L. Hamadeh H. Bushel P.. (2001). Assessing gene significance from cDNA microarray expression data via mixed models. J. Comput. Biol. 8, 625–637. 10.1089/106652701753307520 11747616

[B53] Wright G. J. Rayner J. C. (2014). *Plasmodium falciparum* erythrocyte invasion: combining function with immune evasion. PLoS Pathog. 10:e1003943. 10.1371/journal.ppat.1003943 24651270 PMC3961354

[B54] Yu T. Park Y. Johnson J. M. Jones D. P. (2009). apLCMS - adaptive processing of high-resolution LC/MS data. Bioinformatics 25, 1930–1936. 10.1093/bioinformatics/btp291 19414529 PMC2712336

[B55] Zhang S. J. Liu C. J. Yu P. Zhong X. Chen J. Y. Yang X.. (2014). Evolutionary interrogation of human biology in well-annotated genomic framework of rhesus macaque. Mol. Biol. Evol. 31, 1309–1324. 10.1093/molbev/msu084 24577841 PMC3995340

