# Supplementary material for: Comparative transcriptomics and metabolomics in a rhesus macaque drug administration study
Source: Front Cell Dev Biol. 2014 Oct 8;2:54. doi: 10.3389/fcell.2014.00054 (PMC4233942; doi:10.3389/fcell.2014.00054)
Supplement: Supplementary file 3 [file DataSheet3.DOCX]

1. **QT Clustering (R code)**

# This R Code is based on algorithm and Matlab code developed by Misha Koshelev,Montague Laboratory.

# QT clustering algorithm as described in:

#

# Heyer, L. J., Kruglyak, S., Yooseph, S. (1999). Exploring expression

# data: Identification and analysis of coexpressed genes. Genome Research

# 9, 1106–1115.

#

# http://genome.cshlp.org/content/9/11/1106.full

# http://genome.cshlp.org/content/9/11/1106/F5.large.jpg

#

# if two sets A{i} have same cardinality, we pick first one

# our distance metric is jackknife correlation

#

# input:

# G-nxp data to cluster (feature * obs)

# d-diameter threshold

# lb-minimum # of genes for each cluster

#

# output:

# idx-nx1 vector containing cluster indices

myCor=function(x,y)

{

corcoef=cor(x,y)

Result=matrix(1,2,2)

Result[1,2]=corcoef

Result[2,1]=corcoef

return(Result)

}

QTCluster_JackKnife=function(G,d,lb)

{

n=dim(G)[1]

p=dim(G)[2]

if (n<=1)

{

idx=1

return(idx)

}

#Compute Jackknife correlation for data set G

J = Inf*matrix(1,n, n) ;

for (i in 1:(n-1))

{

J[i,i] = 1 ;

for (j in (i+1):n)

{

for (mm in 1:p)

{

p_del = 1:p ;

p_del= p_del[-mm] ; # obs. deletion

# pearson correlation with one obs. deleted

corr_del = myCor(as.numeric(G[i, p_del]), as.numeric(G[j, p_del])) ;

J[i, j] = min(J[i, j], corr_del[1, 2]) ;

}

# normal pearson correlation

corr_nor = myCor(as.numeric(G[i, ]), as.numeric(G[j, ])) ;

J[i, j] = min(J[i, j], corr_nor[1, 2]) ;

J[j, i] = J[i, j] ;

}

}

J[n,n]=1

# QT_clust procedure

# record for final cluster

C=NULL;

Ccard=0;

Cdiam=0;

for (i in 1:n)

{

flag = T ;

A = i ;

Acard = 1 ;

Adiam = 0 ;

while (flag && length(A)< n)

{

# remaining set

p_remain = 1:n ;

p_remain=p_remain[-A];

jdiam = matrix(0,length(p_remain), 1) ;

for (jj in 1:dim(jdiam)[1] )

{

# we only need to compute minimal distance from new point j to

# all existing points in cluster A

jdiam[jj] = 1-min(J[p_remain[jj], A]) ;

}

# always pick the first met minimal first

min_jdiam = min(jdiam)

min_idx = which (jdiam ==min(jdiam))

# modified by WWY on Mar/25/2014: always pick the first met

# minimal if there exists more than one minimal

if (max(Adiam, min_jdiam) > d )# diameter(A U{j}) exceeds predefined d

{flag = F ;} # stop clustering

else

{# update current set by adding j

A =c(A, p_remain[min_idx]) ;

Acard = Acard + 1 ;

Adiam = max(Adiam, min_jdiam) ;

}

}

# without sorting A

if (Acard>Ccard )

{

C = A ;

Ccard = Acard ;

Cdiam = Adiam ;

}

}

# if the numbers of current largest cluster contains is less than

# predefined number of minimum number

# algorithm stops

if (Ccard < lb)

{

idx=Inf*matrix(1,n, 1)

return(idx) ;

}

else

{

idx=matrix(1,n, 1)

GmC=1:n;

GmC=GmC[-C]

idx[GmC]=qtclustjackknife(G[GmC,],d, lb)

idx[GmC]=idx[GmC]+1;

return(idx)

}

}

# Main Program to call QTCluster_JackKnife

DataFile=dir(".",".csv")

G=read.csv(DataFile)

G=as.matrix(G[,-1]) #Column 1 is the feature ID

d=0.3

lb=2

Result=QTCluster_JackKnife(G,d,lb)

write.csv(Result, paste("Cluster Result_",DataFile,sep=""))

1. **Stochastic Discretization (Matlab)**

function sd = stochasticDiscretizationMatrix(observationVec, discPolicyVec)

% code implementing stochastic discretization.

numObserv = length(observationVec) ;

numLevels = length(discPolicyVec) -1 ;

sd = zeros(numObserv, numLevels) ;

for ii = 1:numObserv

currObserv = observationVec(ii) ;

currMean = currObserv.*ones( numObserv, 1) ;

y = sqrt(sum((observationVec - currMean).^2)*(1/(numObserv-1))) ;

normCumDist = normcdf(discPolicyVec, currObserv, y) ;

sd(ii, :) = normCumDist(2:numLevels+1) - normCumDist(1:numLevels) ;

end

end

1. **Discretization Coalescence (Matlab)**

function coalescenceInfo = timDiscretizationLevelCoalescence(arrayOfdiscMatrics, finalLevels)

% Code implementing the informative-preserving discretization level

% coalescence algorithm.

% Input: arrayOfdiscMatrics is the discretization matrics, it is N*D, N is

% the number of observation, and D is the level degree.

% Ouput: coalescenceInfo records how much mutual information was lost and

% which levels were meaged in each round

numVar = length(arrayOfdiscMatrics) ;

[numObserv, numLevels] = size(arrayOfdiscMatrics{1}) ;

numDLC = numLevels - 1 ;

coalescenceInfo = zeros(numDLC, numVar, 2) ;

if finalLevels >= numDLC

disp('Initial discretization levels are less than the final levels!') ;

return ;

end

for i = 1:(numDLC-finalLevels+1)

for j = 1:numVar

currNumLevels = numLevels - i ;

sumI = zeros(1, currNumLevels) ;

for k = setdiff(1:numVar, j)

Px = sum(arrayOfdiscMatrics{j})/numObserv ;

Py = sum(arrayOfdiscMatrics{k})/numObserv ;

Pxy = (arrayOfdiscMatrics{j}'*arrayOfdiscMatrics{k})/numObserv ;

Hx = -Px.*(log(Px)/log(2)) ;

Hy = -Py.*(log(Py)/log(2)) ;

Hxy = -Pxy.*(log(Pxy)/log(2)) ;

rowIx = Hx - nansum(Hxy') ;

I = nansum(rowIx)+nansum(Hy) ;

tPx = Px(1:currNumLevels) + Px(2:currNumLevels+1) ;

tPxy = Pxy(1:currNumLevels, :) + Pxy(2:(currNumLevels+1), :) ;

tHx = -tPx.*(log(tPx)/log(2)) ;

tHxy = -tPxy.*(log(tPxy)/log(2)) ;

tI = nansum([repmat(I, [1, currNumLevels]); -rowIx(1:currNumLevels); -rowIx(2:currNumLevels+1); (tHx-nansum(tHxy'))]) ;

sumI = sumI + tI ;

end

[maxMutualInfo, maxId] = max(sumI) ;

coalescenceInfo(i, j, :) = [maxId, maxMutualInfo] ;

end

for j = 1:numVar

coalesceIdx = coalescenceInfo(i, j, 1) ;

currDiscMatrix = arrayOfdiscMatrics{j} ;

currDiscMatrix(:, coalesceIdx) = currDiscMatrix(:, coalesceIdx) + currDiscMatrix(:, coalesceIdx+1) ;

currDiscMatrix(:, coalesceIdx+1) = [] ;

arrayOfdiscMatrics{j} = currDiscMatrix ;

end

end

coalescenceInfo=(arrayOfdiscMatrics)

end

1. **Interval Policy Vector (Matlab)**

function ipv = intervalPolicyVector(observationVec, numLevels)

% code implementing the creation of an interval discretization policy

% vector.

% input: observationVec: N observations for one variable x

% numLevels: a discretization policy of degree

% output: ipv: the interval policy vector (the boundary vector)

sortedObserv = sort(observationVec) ;

ipv = zeros(1, numLevels-1) ;

for j = 1:(numLevels-1)

bndrySpacing = (sortedObserv(end)-sortedObserv(1))/numLevels ;

ipv(j) = sortedObserv(1) + (j*bndrySpacing) ;

end

ipv = [-inf, ipv, inf] ;

end
